# Combining genotyping approaches improves resolution for association mapping: a case study in tropical maize under water stress conditions

**DOI:** 10.3389/fpls.2024.1442008

**Published:** 2025-01-23

**Authors:** Fernanda Carla Ferreira de Pontes, Ingrid Pinheiro Machado, Maria Valnice de Souza Silveira, Antônio Lucas Aguiar Lobo, Felipe Sabadin, Roberto Fritsche-Neto, Júlio César DoVale

**Affiliations:** ^1^ Postgraduate Program of Plant Science, Federal University of Ceará, Fortaleza, Ceará, Brazil; ^2^ College of Agriculture and Applied Sciences, Utah State University, Logan, UT, United States; ^3^ Louisiana State University Agricultural Center, Baton Rouge, LA, United States

**Keywords:** SNP-array, genotyping by sequencing, simulated genome, GWAS, genotyping platforms, candidate genes, genes with similar functions

## Abstract

Genome-wide Association Studies (GWAS) identify genome variations related to specific phenotypes using Single Nucleotide Polymorphism (SNP) markers. Genotyping platforms like SNP-Array or sequencing-based techniques (GBS) can genotype samples with many SNPs. These approaches may bias tropical maize analyses due to reliance on the temperate line B73 as the reference genome. An alternative is a simulated genome called “Mock,” adapted to the population using bioinformatics. Recent studies show SNP-Array, GBS, and Mock yield similar results for population structure, heterotic groups definition, tester selection, and genomic hybrid prediction. However, no studies have examined the results generated by these different genotyping approaches for GWAS. This study aims to test the equivalence among the three genotyping scenarios in identifying significant effect genes in GWAS. To achieve this, maize was used as the model species, where SNP-Array genotyped 360 inbred lines from a public panel via the Affymetrix platform and GBS. The GBS data were used to perform SNP calling using the temperate inbred line B73 as the reference genome (GBS-B73) and a simulated genome “Mock” obtained *in-silico* (GBS-Mock). The study encompassed four above-ground traits with plants grown under two levels of water supply: well-watered (WW) and water-stressed (WS). In total, 46, 34, and 31 SNP were identified in the SNP-Array, GBS-B73, and GBS-Mock scenarios, respectively, across the two water levels, associated with the evaluated traits following the comparative analysis of each genotyping method individually. Overall, the identified candidate genes varied along the various scenarios but had the same functionality. Regarding SNP-Array and GBS-B73, genes with functional similarity were identified even without coincidence in the physical position of the SNPs. These genes and regions are involved in various processes and responses with applications in plant breeding. In terms of accuracy, the combination of genotyping scenarios compared to those isolated is feasible and recommended, as it increased all traits under both water conditions. In this sense, it is worth highlighting the combination of GBS-B73 and GBS-Mock scenarios, not only due to the increase in the resolution of GWAS results but also the reduction of costs associated with genotyping and the possibility of conducting genomic breeding methods.

## 1 Introduction

Water is the most abundant and often limiting of all plant resources needed to grow and function (Taiz et al., 2015). Water availability is considered one of the most influential factors in agricultural productivity, controlling species distribution in different climatic zones on Earth (Turner and Jones, 1980). In the tropical zone, characterized by relatively high temperatures and low rainfall compared to other zones, plants thriving in these environments are often more exposed to prolonged periods of water scarcity, especially in arid and semi-arid regions. According to climate change projections, this scenario will likely continue or worsen over the years, with potentially more drastic effects on plants (Raza et al., 2019; IPCC, 2023).

Stress can be considered a significant deviation from optimal life conditions (Larcher, 2003), inducing changes and responses as the plant fails to complete its physiological processes for growth and production. The lack of adequate water supply causes greater expansion of the root system into deeper and moister zones of the soil profile, reduction in the development of cells in the aerial tissues, resulting in decreased growth and stomatal closure to reduce transpiration rate and, consequently, photosynthetic activity (Frensch and Hsiao, 1994; Hsiao, 1973). Control measures are complex and difficult for humans to manage, and the search for genotypes that will perform better and economically viable yields in water-limited environments has been increasingly important for genetic improvement.

Conventional breeding for water deficit conditions is still time-consuming, laborious, and costly, as experimental conditions must be carefully managed. However, in recent years, with advances in molecular biology, the development of high-throughput genotyping technologies, and progress in platform development, new opportunities have emerged to enhance this process. This is partly due to cost reduction, which has consequently driven advances in genomic sequencing; another factor is the versatility of SNP (Single Nucleotide Polymorphism) markers, most commonly used in this process (Ingvarsson and Street, 2011). SNPs are abundant markers in crop genomes and are ideal for genetic discovery research and molecular improvement (Rasheed et al., 2017). According to the same authors, genotyping platforms involving Next Generation Sequencing (NGS) and SNP-Array technologies are suitable for genotyping hundreds to thousands of samples with many SNP markers in a single assay much more quickly, revolutionizing the study of genomics and molecular biology.

Genotyping techniques by sequencing or GBS (Genotyping by Sequencing) are simple and highly multiplexed systems used for constructing libraries intended for next-generation sequencing. SNP-Array is a technique that uses microarrays designed to pre-select previously identified genetic markers characterized by wide polymorphism. These markers are then incorporated into a specific platform. GBS-scored SNP platforms provide many markers, although they have high rates of missing data. On the other hand, Array-scored SNP platforms are of high quality but have relatively high costs (Elbasyoni et al., 2018) and possible ascertainment bias if the genetic material used for array development is not related to the tested germplasm (Heslot et al., 2013).

Arrays are well-designed and established in the market to assist studies and breeding programs of major commodity crops (Ganal et al., 2011). For minor crops, arrays are still rarely available, and researchers often rely on information from other crops that is already accessible. However, due to the high cost associated with array development, these platforms are preferably employed when it is possible to use a “universal” approach that applies to various germplasms. However, this can be challenging if researchers attempt to identify rare SNPs across various germplasms; a universal design can become large and expensive, resulting in many monomorphic loci for non-target germplasm groups (Thomson, 2014).

The advancement of model genome knowledge and the advent of next-generation sequencing techniques open up the possibility of a great leap in understanding the genome of relatively lesser-known species. The GBS pipelines are based on a reference genome or assembly of a new genome, applied to model organisms and species lacking pre-existing genomic information (Davey et al., 2011; Poland et al., 2012). In cases where a reference genome is not yet available, a simulated genome can be employed for SNP discovery, which can serve as a valid alternative (Melo et al., 2016). The same authors developed a bioinformatics pipeline to construct a simulated genome called “Mock,” adapted to the population and built from GBS data. This genome is already being used in genomic studies and indicated that the Mock produces similar results when it comes to organizing populations, identifying heterotic groups, selecting testers, and predicting genomic characteristics of hybrids compared to standard approaches (SNP-Array and GBS) (MaChado et al., 2023; Sabadin et al., 2022). This suggests that simulated genomes can be a good alternative, especially for species without the reference genome. However, no studies have been identified on the results generated by these different genotyping approaches in Genome-Wide Association Studies (GWAS).

Other studies have compared datasets from different high-throughput genotyping technologies in GWAS. Darrier et al. (2019), using standard platforms, GBS and SNP-Array, demonstrated efficiency in characterizing genetic diversity in barley, although accessing different regions of the genome. Despite capturing different areas, there was a positive correlation between the genetic distance matrices of both approaches, validating the use of either one for the characterization. These authors emphasized that the choice between GBS and SNP-Array genotyping platforms should be based on various factors, including the nature of the research and group preferences. For example, GBS may be preferable for studies requiring broader genomic coverage due to its ability to sequence a large number of genetic markers.

Conversely, SNP-Array may be more appropriate for analyses focused on specific genome regions. Group preferences, previous experience, and practical considerations such as cost and resource availability influence platform choice. In a study with inbred maize lines, Negro et al. (2019) concluded that GBS and SNP-Array were complementary for detecting QTL marking different haplotypes in association studies. Assuming they are complementary, combining these platforms seeks to determine if it will result in greater data accuracy.

To date, no study comparing GBS, SNP-Array, and simulated genome for GWAS has been published yet. The application of studies of this nature is crucial because they provide evidence that the information obtained from various genotyping approaches may be complementary during the genotyping process, thus demonstrating an efficient alternative for identifying polymorphisms. This, in turn, should offer better support to breeding programs that consistently grapple with identifying more efficient and tolerant genotypes against various abiotic and biotic factors. Another relevant point is that, even with advances in whole-genome sequencing and the complete publication of the maize genome, approaches such as SNP-Array and GBS remain important due to their lower cost and efficiency in genotyping large populations. These techniques generate more manageable data, requiring less computational infrastructure, and provide sufficient resolution to address many biological questions. Their effectiveness in GWAS studies and identifying loci in crops like maize is well demonstrated. For breeding programs or projects with limited resources, they represent agile and viable alternatives, balancing cost, accessibility, and quality.

In this context, the objectives of this study were: i) to verify if there is a difference in the identification of genes with significant effects among genotyping platforms, SNP-Array, GBS, and simulated genome (“Mock”) in GWAS; ii) once differences are confirmed, to determine if the identified genomic regions are complementary and if they provide better accuracy.

## 2 Materials and methods

**Figure 1:**
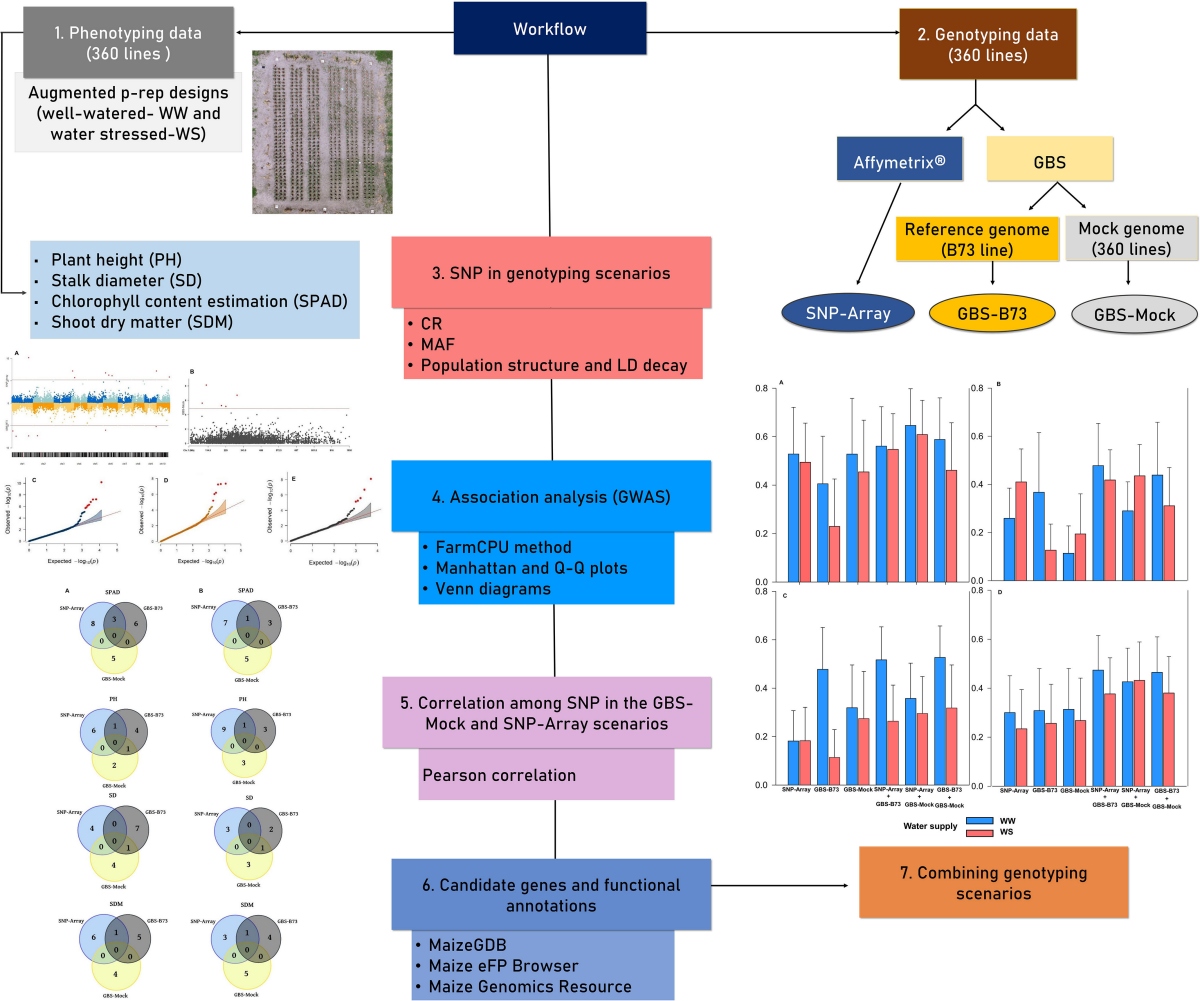
The workflow employed in the study. Different colors are used to represent distinct phases of the analysis.

To enhance the comprehensibility of the analyses conducted in this study, we present a workflow in which the experimental and data analysis components are summarized in **Figure 1**. The subsequent sections provide detailed explanations.

### 2.1 Genetic material and experimental trials

This study used maize as the model species in a public diversity panel of 360 tropical inbred lines (Yassue et al., 2021). The genomic and phenotypic data of this panel can be found on the Mendeley platform (https://data.mendeley.com/datasets/6pb9prrbbb/1). The data to be explored were obtained from eight experiments conducted in 2020 and 2021, as detailed below. This study involves contrasting water supply conditions, well-watered (WW) and water-stressed (WS), so a pilot experiment was conducted before the main experiments. A water retention curve was established through regression to obtain field capacity and determine the amount of water to be provided via irrigation (De Souza Silveira et al., 2024). This pilot experiment involved five randomly selected lines from the panel and five levels of water supply: 100% of water applied (WA), 80% of WA, 70% of WA, 50% of WA, and 40% of WA. As a result, the WW and WS points were determined, with the 80% WA and 40% WA treatments representing these conditions, respectively.

The main experiments were conducted at experimental fields of the Department of Agriculture at UFC, Campus do Pici, Fortaleza-CE, located at 3°44’24.27” S latitude and 38°34’29.93” W longitude. The main experiments were conducted under WW and WS in augmented partially repeated block design (augmented p-rep designs), with two temporally spaced replicates (Williams et al., 2011). Five common treatments (checks) were used, randomly selected from within the panel and distributed in each block within the WW and WS conditions (**Supplementary Figure S1**).

These experiments were always conducted in the second semester of each year, following the rainy season in the region, a period that resembles the climate of the semi-arid zone. The sowings were carried out in plastic pots with a capacity of 2000 cm^3^, containing substrate (easily reproducible) in a ratio of 3:1 (sand: earthworm humus). The earthworm humus was chosen due to its easy obtainability and effectiveness in providing nutrients to the plants. The use of sand is justified by its easy acquisition, availability, and low cost.

Two seeds were sown per pot at an average 3-4 cm depth. Thinning was performed when the seedlings reached the V2 stage, leaving only one seedling per pot (plot). At this same phenological stage, a water deficit was also initiated, which continued until the V6 stage (harvest). Planting and topdressing fertilization were based on the chemical analysis of the substrate, taking into consideration the crop recommendations, to isolate nutritional stress during the experimental conduct.

As the experiment was conducted in an open field, irrigation control for each experiment was carried out manually and daily. Thus, 15 random samples were used to calculate the daily average weight of the pots within each water supply level. Subsequently, the difference between the current and total weights obtained at each water supply level was calculated to replenish the water volume. It is worth noting that, for each vegetative stage, the average plant weight was obtained to subtract it along with the current weight, thus not affecting the volume of water to be replenished.

### 2.2 Phenotypic data

The phenotypic evaluation was conducted when most plants reached the V6 phenological stage. The traits considered in this study were: Plant height (PH) - measured from the soil to the insertion of the flag leaf, measured using a graduated ruler (cm); Stalk diameter (SD) – an average of two measurements above ground level at the second node of the stem obtained using a caliper (mm); Chlorophyll content estimation - using SPAD, measuring three leaves per plant to get the average. Subsequently, the plants were cut off at ground level, placed in paper bags, and put in a forced-air oven at 65°C for 72 hours to obtain: Shoot dry matter (SDM)- quantified using an electronic analytical balance (0,005 g).

### 2.3 Phenotypic analysis

The outliers of the phenotypic data for the traits described in section 2.2 were removed. Then, the remaining data were adjusted for normality using the *bestNormalize* package (Peterson, 2021), and the assumptions of normal distribution were checked via the Shapiro test and Q-Q *plots*. Subsequently, equations of mixed linear models were fitted to obtain the BLUP by REML for each trait studied under WW and WS conditions, using the *sommer* package (Covarrubias-Pazaran, 2016). These analyses were performed using the following model:


(1) $$\text{y}={\text{X}}_{1}\text{t}+{\text{X}}_{2}\text{l}+{\text{X}}_{3}\text{n}+{\text{Z}}_{1}\text{b}+{\text{Z}}_{2}\text{g}+{\text{Z}}_{3}\text{i}+\text{ϵ}$$

where, *y* is the vector of phenotypic values of the inbred lines panel and checks; X_1_, X_2_, and X_3_ are incidence matrices for *t*, *l*, and *n* fixed effects; Z_1_, Z_2_ and Z_3_ are incidence matrices for *b*, *g* e *i* random effects; *t* is the water supply fixed effect vector (WW and WS conditions); *l* is the replicate (season) fixed effect vector within water supply; *n* is the number of leaves used as a covariate to correct for differences in plant vigor and development; *b* is the block/water supply/season random effect vector, where g~N(0, $I\sigma_{b}^{2}$ ); *g* s the genotype random effect vector, where g~*N*(0, $I\sigma_{g}^{2}$ ); *i* is the random effect vector of the genotype–water supply interaction, where i~*N* (0, $I\sigma_{i}^{2}$ ); ε is the experimental error, where ε~*N*(0, $R\sigma_{e}^{2}$ ), obtained using a structured diagonal matrix to make it possible to estimate two residual variances, one for each water supply level ( $\sigma_{eWW}^{2}$ and $\sigma_{eWS}^{2}$ ). The significance of fixed effects was assessed using the Wald test, and random effects using the likelihood ratio test.

The variance components were used to estimate the heritabilities (*h^2^*) by the following estimator:


(2) $${\text{h}}^{2}=\frac{{\text{σ}}_{g}^{2}}{{\text{σ}}_{g}^{2}+\frac{{\text{σ}}_{\text{ge}}^{2}}{\text{s}}+\frac{\left({\text{σ}}_{e\text{WW}}^{2}+{\text{σ}}_{e\text{WS}}^{2}\right)}{\text{rs}}}$$

where *h* ^2^ refers to the entry-mean heritability; $\sigma_{g}^{2}$ is the genotypic variance of the inbred lines panel, $\sigma_{ge}^{2}$ is the variance of the genotype–water supply interaction; $\sigma_{eWW\;e\;}^{2}\sigma_{eWS}^{2}$ are the environmental variance components in WW and WS; *s* are levels of WW and WS; and *r* is the number of repetitions in each water supply level.

The reliability of selection for each line [ $R^{2}({\hat{\alpha }}_{l})$ was obtained by the following expression (Gorjanc et al., 2015):


(3) $$R^{2}({\hat{\alpha }}_{l})=1-\frac{Var(\alpha_{i}-{\hat{\alpha }}_{i})}{Var(\alpha_{i})}$$

where $Var(\alpha_{i}-{\hat{\alpha }}_{i})$ is the variance of the prediction error (PEV) of line *i* and $Var(\alpha_{i})$ is the genotypic variance of the trait.

The de-regressed BLUPs (dBLUPs) were obtained by calculating the ratio between the BLUPs of each inbred line in WW and WS and their respective average reliabilities. After these analyses, 313 lines remained out of the 360 in the panel. The dBLUPs of these lines in WW and WS were used in the GWAS analyses.

### 2.4 Genotypic data

The lines were genotyped using two SNP genotyping platforms: Affymetrix^®^ Axiom Maize Genotyping Array with 18.413 SNP markers (SNP-Array) and genotyping-by-sequencing (GBS) process following the sequencing protocol established by Poland et al. (2012). In this method, genomic DNA was digested by two restriction enzymes, PstI and MseI, to reduce the genome complexity. Subsequently, specific adapters for sequencing on the Illumina NextSeq 500 platform (Illumina Inc., San Diego, CA, United States) were attached to the digested fragments.

The primary GBS data were employed for two purposes: firstly, to perform SNP calling using the temperate line B73 as the reference genome (RefGen v4). Secondly, to construct a simulated reference genome (mock genome) for SNP calling, following the pipeline proposed by Melo et al. (2016), considering all the lines in the panel (Mock).

Therefore, the SNP data were subjected to three GWAS approaches: 1) SNP-Array; 2) GBS with SNP calling based on the B73 reference genome (GBS-B73); 3) GBS using the simulated genome as the reference (GBS-Mock). The SNPs for the GBS dataset was identified from raw data using the TASSEL 5.0 GBSv2 pipeline (Glaubitz et al., 2014), considering both GBS-B73 and GBS-Mock as reference genomes, employing the BWA aligner. The BWA aligner (Li and Durbin, 2009) was used to align the tags against the reference genome (GBS-B73 and GBS-Mock).

The SNP sets obtained in these scenarios were submitted to quality control parameters as call rate (CR) and Minor Allele Frequency (MAF) procedures, where markers with CR < 90% and MAF lower than 5%, and non-biallelic markers were removed from the datasets (Morosini et al., 2017). Imputation of missing data was performed using the Beagle 5.0 algorithm (Browning and Browning, 2008).

### 2.5 Population structure and LD decay

In order to minimize potential bias caused by population structure, a PCA was performed based on the additive genomic relationship matrix among the remaining 313 panel lines, following VanRaden (2008) using the *SNPRelate* package (Zheng et al., 2012). FarmCPU automatically incorporated the correction via PCA in the association analysis. Two principal components were used to correct the population structure effect, and the best fit for the model was determined based on Q-Q *plots*. The most likely number of groups within the panel was determined according to Yassue et al. (2021) as it involved the same diversity panel.

The Linkage Disequilibrium (LD) estimation between each pair of SNP within the chromosomes was calculated by the square of the allele frequency correlation (r²) among all SNP within a distance less than 1 Mbp. The r² values were plotted against the base pair distance of the SNP pair to obtain the LD decay by chromosome. This procedure was performed with all SNP retained from the quality control procedures.

### 2.6 Association analysis

GWAS were performed for each trait under WW and WS conditions using the FarmCPU method (Liu et al., 2016a). The method stands out for its computational efficiency and ability to control false positives, demonstrating greater statistical power in situations where the trait is strongly associated with kinship (Liu et al., 2016a; Segura et al., 2012). The *FarmCPU.P.Threshold* function was employed to obtain the *p-threshold*, specific for each trait via a simulation process with 100 permutations. Subsequently, the cutoff point was obtained by the ratio between the *p-threshold* and the number of markers used. Subsequently, p-values (significance), MAF, and ASE (Average Effect of Allele Substitution) were obtained for each significantly associated SNP, designated hereafter as a potential candidate gene underlying the target trait. Furthermore, the coefficient of determination for each significant SNP ( $R_{SNP}^{2}$ was obtained based on ASE and MAF using equations described in Da et al. (2014). Next, multiple linear regressions were established for each trait using the significant SNPs as predictor variables to quantify the markers’ influence on that trait’s expression ( $R_{TOT}^{2}$ ). The Manhattan and Q-Q *plots* graphs were generated using the CMplot package (Yin, 2020), and the graphs showing the proportion of phenotypic variance explained by the SNP were generated using the ggplot2 package (Wickham, 2011) in the R software. Venn diagrams based on the common gene functionality for the traits at each water supply level were created using LucidChart (lucidchart.com).

### 2.7 Correlation among markers of different scenarios

Given the stability and efficiency of SNP-Array technology in accurately genotyping numerous markers, we conducted Pearson correlation analysis (r) among significant markers with known functions identified in GWAS within the GBS-Mock scenario and markers present in the SNP-Array scenario for each trait under both WW and WS conditions. The analyses were performed using the R software base. This approach aimed to assess the concordance and potential overlap between markers identified through different genotyping methods and their associations with specific traits. By comparing these markers across scenarios, we sought to elucidate common genetic factors contributing to trait variation and explore the utility of integrating data from diverse genotyping platforms in genomic analyses related to crop improvement and adaptation to environmental stressors.

### 2.8 Gene annotation

A candidate gene association mapping was performed for traits with significant SNP. The physical positions of SNP for GBS-Mock were assigned using BLAST (Altschul et al., 1990) to align them with the maize genome assembly for comparison purposes. These positions were used to obtain 41 bp DNA fragments on a single chromosome (**Supplementary Table S2**). Subsequently, a BLAST was conducted exclusively for GBS-Mock on MaizeGDB^1^ via blast, utilizing the B73 RefGen_v4 sequence database to locate the chromosome by inserting the DNA fragment. The MaizeGDB database and its functional information associated with each SNP based on B73 RefGen_v4 were utilized for all scenarios. After defining the region to be considered, potential candidate genes flanking each marker were identified. Candidate genes linked to each trait were determined through annotation within a sliding window of 50 kb around each significant SNP, following a conservative approach described by Yassue et al. (2021). All genes within a range of 50 kb downstream and 50 kb upstream were annotated. Subsequently, they were assessed and considered based on two criteria: proximity to the SNP and functional similarity as per databases available on the Maize eFP Browser (2023)^2^ and Maize Genomics Resource (2023)^3^.

## 3 Results

### 3.1 Phenotypic analysis

In general, significant effects were detected for all sources of variation, except for the G x WA interaction, in the studied traits (**Table 1**). The variance components showed a similar pattern for all traits, with a predominance of genotypic variance over the residual variance of the interaction. Except for the PH trait, there was a higher residual variance for the well-watered environment than the low-water availability. The genotypic variance component ranged from 0.09 to 0.19, and the genotype x environment interaction approached zero for all traits, affecting the estimates of heritabilities and accuracy. Heritabilities ranged from moderate to high magnitude, ranging from 0.58 to 0.73. PH was the trait with the highest heritability (0.73) and the least influenced by the environment, showing the highest genotypic coefficient of variation (0.197). The adjusted means fall within the same range observed in other studies.

**Table 1:** Wald test of fixed effects, likelihood-ratio test (LRT) of random effects, variance components, heritability, accuracy, and adjusted average for SPAD, plant height (PH), stalk diameter (SD), and shoot dry matter (SDM) of the inbred lines evaluated in WW (well-watered) and WS (water-stressed) conditions water supply.

Source of variation	SPAD	PH	SD	SDM
	Wald statistic			
Water supply (WA)	1098.27***	4353.03***	4549.80***	5393.32***
Replicates/WA	1076.83***	2542.48***	1842.71***	2462.96***
	Likelihood-ratio test (LRT)			
Block/WA/Season	89.35***	40.76***	46.21***	212.23***
Genotypes (G)	102.59***	201.98***	100.60***	96.35***
G x WA	1.11^NS^	1.08 ^NS^	0.72 ^NS^	2.43 ^NS^
	Variance components			
$\sigma_{g}^{2}$	0.192	0.197	0.119	0.099
$\sigma_{g\times e}^{2}$	0.015	0.007	0.007	0.012
$\sigma_{eWW}^{2}$	0.525	0.266	0.352	0.299
$\sigma_{eWS}^{2}$	0.511	0.279	0.275	0.240
	Heritability and accuracy			
*h* ^2^	0.58	0.73	0.59	0.58
	Adjusted means			
	31.13	9.44	7.56	2.32

***significant at the 0.001 probability level (by Wald test or LRT), respectively. ^NS^non-significant.

### 3.2 Genotypic scenarios: number and distribution of SNP

**Figure 2:**
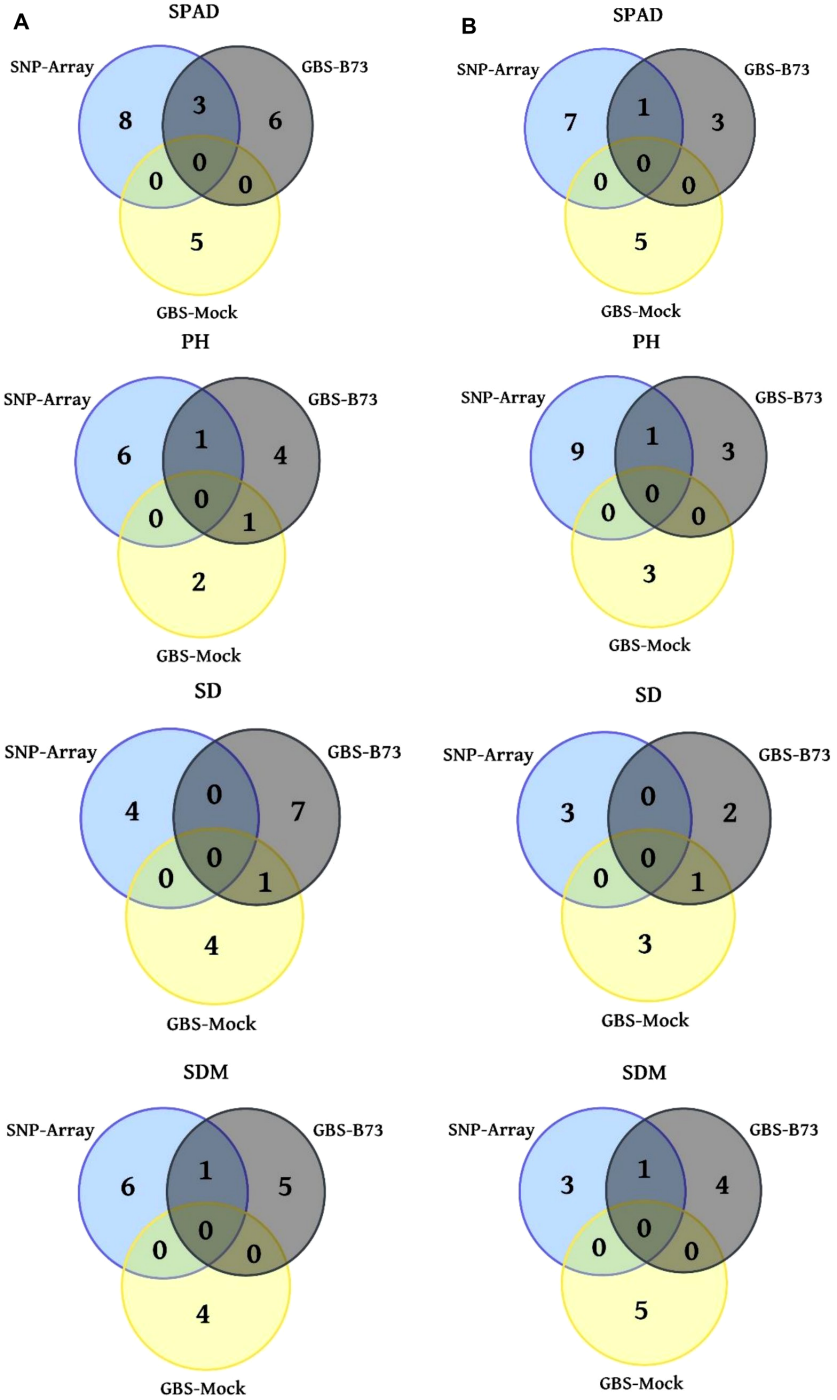
Venn diagrams with the number of significant trait SNPs in three genotyping scenarios. **(A)** WW (well-watered) water supply condition column; **(B)** WS (water-stressed) water supply condition column. SPAD, PH (plant height), SD (stalk diameter), and SDM (shoot dry matter).

After the quality control, heterozygous markers were eliminated using the MAF and CR procedures, resulting in 12.704 SNP markers for SNP-Array out of a total of 18.413, 11.153 out of 131.350 for GBS-B73, and 4.935 out of 46.926 for GBS-Mock, which were used in the association analyses (**Table 2**). Approximately 69% of the marker set remained in the SNP-Array, while 10.5% remained in the GBS-Mock and 8.5% in the GBS-B73 scenario. However, there was a balanced distribution of SNP across the chromosomes in the standard scenarios (SNP-Array and GBS-B73). In total, 11 common SNPs were found among the genotypic scenarios (**Figure 2**). Between SNP-Array and GBS-B73, 8 shared SNPs were observed: 3 for SPAD under well-watered conditions and 1 under water-stressed conditions; 1 SNP in each water supply condition for PH and SDM. In the GBS-B73 and GBS-Mock scenarios, 1 common SNP was identified for PH under well-watered conditions and 1 for SD in both water supply conditions.

**Table 2:** Number of markers scored (*raw data*) and the final number of markers (*clean data*) total and per chromosome (Chr) after quality control for all genotyping scenarios used to assess inbred lines evaluated in WW (well-watered) and WS (water-stressed) conditions water supply.

	Genotyping scenarios^a^		
	SNP-Array	GBS-B73	GBS-Mock
*Raw data*	18,413	131,350	46,926
*Clean data*	12,704	11,153	4,935
Chrm 1	1,977 (15.6%)	1,651 (15.0%)	Unique chrm
Chrm 2	1,643 (12.9%)	1,411 (12,7%)	
Chrm 3	1,430 (11.3%)	1,269 (11.4%)	
Chrm 4	1,412 (11.1%)	1,177 (10.6%)	
Chrm 5	1,373 (10.8%)	1,336 (12.0%)	
Chrm 6	1,018 (8.0%)	859 (7.7%)	
Chrm 7	957 (7.5%)	831 (7.5%)	
Chrm 8	1,116 (8.8%)	964 (8.6%)	
Chrm 9	973 (7.7%)	855 (7.7%)	
Chrm 10	805 (6.3%)	780 (7.0%)	

a SNP-Array, Affymetrix^®^ Axiom Maize Genotyping array; GBS-B73, genotyping-by-sequence with SNP calling using B73 as reference genome; GBS-Mock, genotyping-by-sequence with SNP calling using the mock reference built with all parental lines.

### 3.3 GWAS analysis

**Figure 3:**
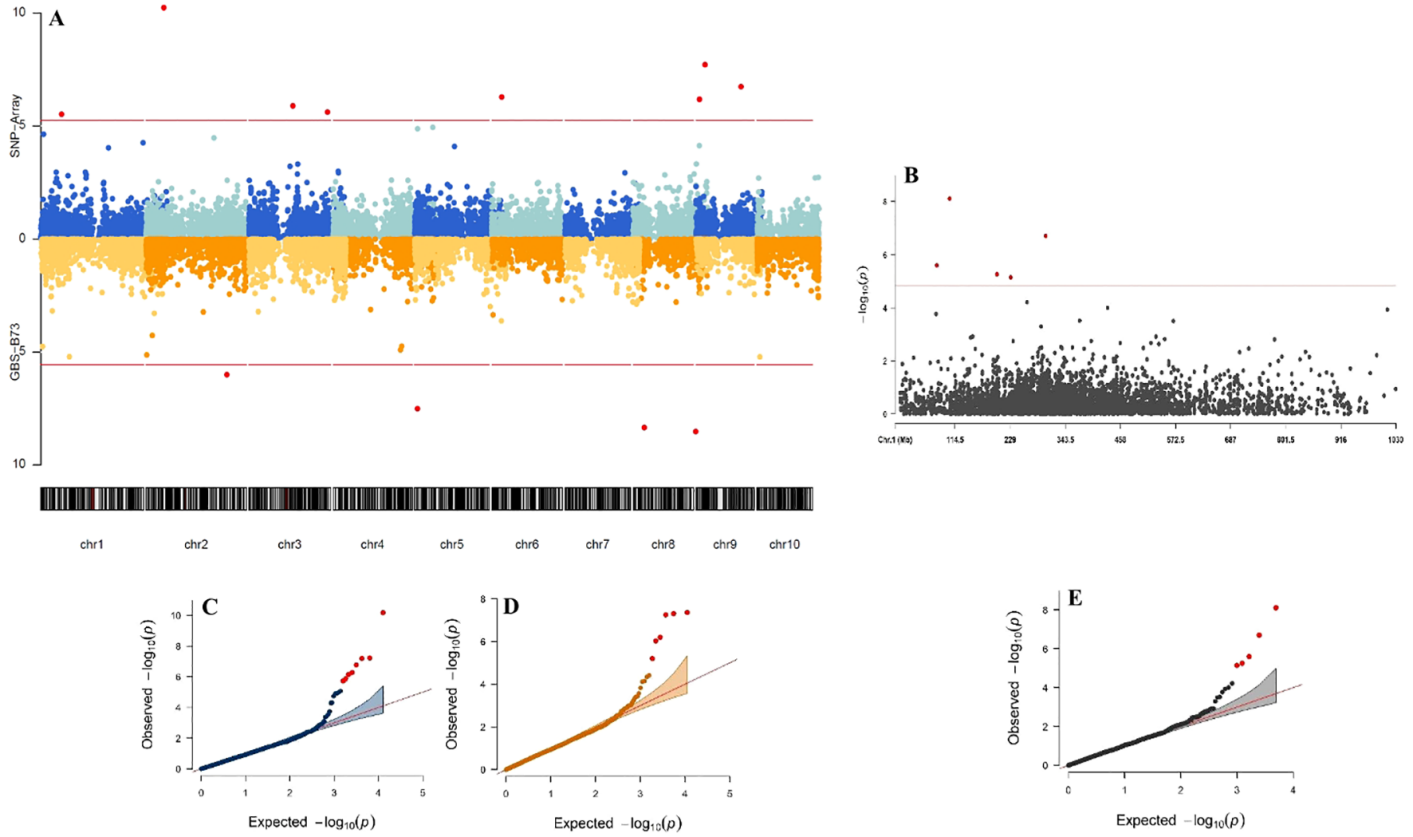
Manhattan plot and Quantile-Quantile (Q-Q) plots for Genome-Wide Association Study (GWAS) comparing genotyping platforms in tropical maize for SPAD trait in WW (water-stressed) conditions water supply. The Manhattan plot displays GWAS results based on three datasets: SNP-Array and GBS-B73 **(A)**, and Mock **(B)**. The x-axis represents the chromosomal positions, while the y-axis indicates the -log10 P-values, reflecting statistical significance. The horizontal lines denote the genome-wide suggestive significance threshold, with dots above these lines marking significant SNPs. The Q-Q plots illustrate the GWAS results for the same datasets: SNP-Array **(C)**, GBS-B73 **(D)**, and Mock **(E)**. The x-axis corresponds to the -log10 expected P-values derived from the chi-square distribution, while the y-axis represents the -log10 observed P-values. Each dot represents an SNP, with the most significant SNP appearing as the top hit. The red diagonal line shows the expected distribution under the null hypothesis of no association.

Significant SNP were found on five of the ten maize chromosomes for the SNP-Array scenario and four for GBS-B73 for the SPAD trait under the WW condition (**Figures 3A, B**). The Q-Q plots showed data fitted to the model (**Figures 3C–E**). The significant marker/trait association threshold ranged from 4.99 to 12.85 (**Supplementary Table S1**).

A total of 46, 34, and 31 significant SNP were found for SNP-Array, GBS-B73, and GBS-Mock, respectively (**Table 3**; **Supplementary Figures S2**–**S9**). There were no SNP common to all three scenarios; however, at least one SNP was shared between two of them (**Figure 2**). SPAD had the highest number of significant SNP, totaling 34, followed by PH, SDM, 27, and SD, 23. The SNP array presented more markers for SPAD and PH and GBS-B73 for SD, and there was an equivalence among the three scenarios for SDM. Overall, GBS-B73 and GBS-Mock showed some similarity in the quantity of markers.

**Table 3:** Number, average, and standard deviation (SD) of significant SNPs per trait in WW (well-watered) and WS (water-stressed) conditions water supply and genotyping scenario.

Water Supply	SPAD			PH			SD			SDM		
	SNP-Array	GBS-B73	GBS-Mock	SNP-Array	GBS-B73	GBS-Mock	SNP-Array	GBS-B73	GBS-Mock	SNP-Array	GBS-B73	GBS-Mock
WW	8	6	5	6	4	2	4	7	4	6	5	4
WS	7	3	5	9	3	3	3	2	3	3	4	5
*Overall*	15	9	10	15	7	5	7	9	7	9	9	9
*Average*	7.5	4.5	5	7.5	3.5	2.5	3.5	4.5	3.5	4.5	4.5	4.5
*SD*	0.5	1.5	0	1.5	0.5	0.5	0.5	2.5	0.5	1.5	0.5	0.5

### 3.4 Correlation among SNP in the GBS-Mock and SNP-Array scenarios

Our results revealed 20 significant markers in the GBS-Mock that positively correlated with the SNP-Array scenario to traits under different environmental conditions (**Table 4**). Pearson correlation coefficients (r) were observed, ranging from weak to strong. Specifically, for SDM in WW conditions, correlations ranged from 0.94 to 0.30. Similarly, SPAD values showed moderate to strong correlations with markers, ranging from 0.52 to 0.76 in WW conditions and from 0.40 to 0.76 in WS conditions. For PH, correlations were moderate, with values of 0.36 for WW and 0.51 and 0.53 for WS. Notably, SD exhibited correlations ranging from 0.35 to 0.87 in WW conditions and from 0.30 to 0.87 in WS conditions. Additionally, SDM showed moderate to strong correlations, ranging from 0.46 to 0.94 in WW conditions and 0.47 in WS conditions.

**Table 4:** Pearson correlation among significant markers from the GBS-Mock scenario with known functions and markers from the SNP-Array scenario for traits in WW (well-watered) and WS (water-stressed) conditions water supply.

Trait	GBS-Mock		SNP-Array		r
	Marker	Position	Chrm	Position	
SPAD in WW	Zm00001d036175	76554426	6	66088433	0.71
			6	68443154	
	Zm00001d042735	201318938	6	76875108	0.76
			6	77806057	
	Zm00001d042755	230114762	1	99951335	0.41
	Zm00001d024497	302570660	10	22670857	0.52
SPAD in WS	Zm00001d008500	200328622	3	179265803	0.40
			4	214338748	
	Zm00001d042735	201318938	6	76875108	0.76
			6	77806057	
	Zm00001d006357	306796326	2	206059268	0.49
	Zm00001d029023	537851163	5	199240894	0.41
PH in WW	Zm00001d012719	305928723	9	1840217	0.36
PH in WS	Zm00001d034057	399525514	1	279982555	0.51
	Zm00001d018703	922415122	8	132436479	0.53
SD in WW	Zm00001d026300	205301556	1	62995980	0.38
	Zm00001d001852	583680097	7	119716831	0.35
	Zm00001d047956	831219654	10	144416974	0.87
SD in WS	Zm00001d001852	583680097	7	119716831	0.35
	Zm00001d053262	751364673	1	33596576	0.30
			1	156697884	
			2	2509863	
			2	176643239	
			2	226012848	
			3	200304796	
			4	30519074	
			4	74777100	
			4	194400472	
			4	195155904	
			4	235373522	
			5	153728179	
			5	219174423	
			7	119716831	
			8	26683716	
			8	136183604	
	Zm00001d047956	831219654	10	144416974	0.87
SDM in WW	Zm00001d046354	320987424	9	84921243	0.94
	Zm00001d008954	511458983	10	94908425	0.46
SDM in WS	Zm00001d018001	440728659	5	211640184	0.47

### 3.5 Candidate genes and functional annotations

Based on the physical location of significant SNP in the B73 reference genome for SNP-Array and GBS-B73 and the reference genome for GBS-Mock, genomic regions and candidate genes related to significant loci were identified (**Supplementary Table S1**). In some cases, the same genes and regions were identified for a given trait under both water supply conditions. For example, *Zm00001d042735* and *Zm00001d001852* in the GBS-Mock scenario for SPAD and SD, respectively; *Zm00001d017978* located on chromosome 5 in SNP-Array for PH. Similarly, identical genes and regions were found in different scenarios, for instance, *Zm00001d031759* located on chromosome 1 was detected in SNP-Array and GBS-B73 for SPAD in WW and WS. The same gene was also identified for different traits, such as *Zm00001d005090* for SD and SDM in GBS-B73.

The genomic regions and candidate genes with similar functions were grouped, considering each trait at the same water supply level across genotyping scenarios (**Figure 2**; **Supplementary Table S3**). For SNP-Array and GBS-B73, regions and genes with the same functionality on the same chromosome were observed, such as *Zm00001d031445* and *Zm00001d027626*, both on chromosome 1, which are correlated with ethylene biosynthesis for SDM in WW. Conversely, these platforms also identified genomic regions and candidate genes on different chromosomes but with coinciding functions. For example, *Zm00001d026477* on chromosome 10 and *Zm00001d027695* on chromosome 1 are responsible for responses to abiotic stress by reactive oxygen species (ROS), jasmonic acid (JA), and ethylene; *Zm00001d044194* on chromosome 3 and *Zm00001d018127* on chromosome 5 function in the regulation of the circadian cycle for SPAD under WW; *Zm00001d017978* on chromosome 5 and *Zm00001d008952* on chromosome 8 are involved in endoglucanase activity for PH in WW; and *Zm00001d053809* on chromosome 4 and *Zm00001d042481* on chromosome 3 for GBS-B73 are associated with ubiquitin proteins for PH in WS; *Zm00001d016786* on chromosome 5 and *Zm00001d005090* on chromosome 2 act in response to water stress through abscisic acid (ABA) for SDM in WS.

In scenarios involving GBS-B73 and GBS-Mock, genomic regions and candidate genes with similar functions were identified for *Zm00001d021708* on chromosome 7 and *Zm00001d012719* on the single chromosome, related to plant responses to ABA for PH in WW; *Zm00001d014899* on chromosome 5 and *Zm00001d001852* on the single chromosome, associated with the phytohormone gibberellin for SD in WW; *Zm00001d00509* on chromosome 2 and *Zm00001d053262* on the single chromosome, involved in ABA regulation for SD in WS.

### 3.6 Phenotypic variation explained by SNP in different genotyping scenarios

**Figure 4:**
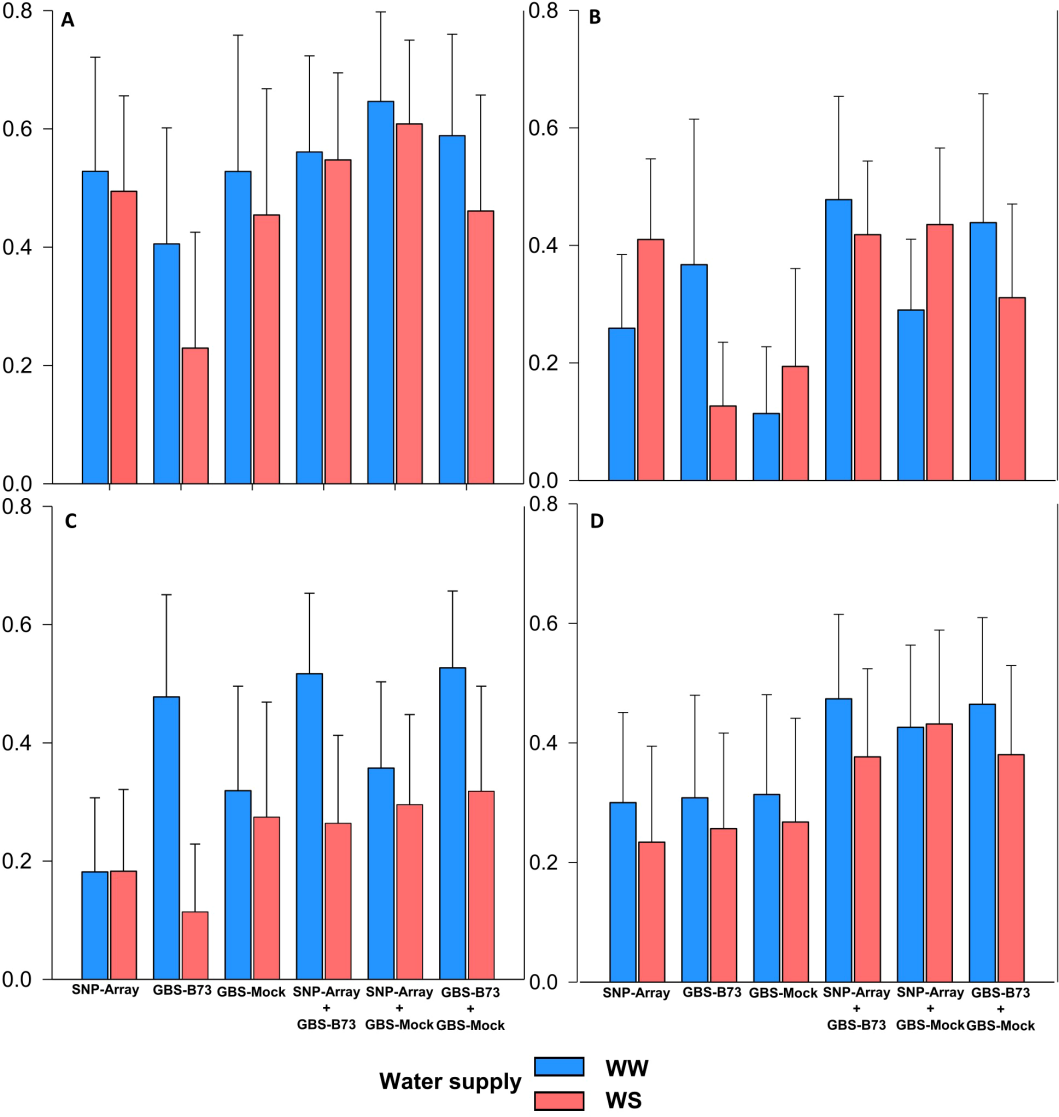
Proportion of phenotypic variance explained by the SNP ( $R_{TOT}^{2}$ ) per trait in WW (well-watered) and WS (water-stressed) conditions water supply and genotyping scenario). **(A)** SPAD, **(B)** PH (plant height), **(C)** SD (stalk diameter), and **(D)** SDM (shoot dry matter).

The proportions of phenotypic variance explained by significant SNP ( $R_{TOT}^{2}$ ) for the analyzed traits under both water supply conditions, ideal (WW) and deficit (WS), were less explained in the isolated genotyping scenarios for the studied traits (**Figure 4**). Regarding the isolated scenarios, $R_{TOT}^{2}$ in SNP-Array ranged from 0.18 for SD (WW in WS) to 0.53 for SPAD (WW), GBS-B73 ranged from 0.11 for SD (WS) to 0.48 for SD (WW), and GBS-Mock from 0.11 for PH (WW) to 0.53 for SPAD (WW). Overall, SNP-Array performed better independently for SPAD and PH, except for SD (WW), where GBS-B73 stood out, and SDM was almost the same among the scenarios. When combined, the value of $R_{TOT}^{2}$ ranged from 0.26 in SNP-Array + GBS-B73 for SD (WS) to 0.65 in SNP-*Array* + GBS-Mock for SPAD (WW). The percentages obtained represent the phenotypic variance explained by combining multiple SNPs simultaneously.

The best scenario combination was SNP-Array + GBS-Mock for SPAD (WW) with an increase of 0.12 in accuracy compared to the best isolated scenario. For PH and SDM under WW condition, SNP-Array + GBS-B73 was superior, increasing accuracy by 0.07 and 0.16, respectively, compared to the best single scenario. For SD, combining GBS-B73 + GBS-Mock increased accuracy by 0.05. Regarding water availability, the ideal water supply condition achieved better overall accuracy, except for PH in isolated SNP-Array and combined with GBS-Mock. In the WS condition, better accuracy was also observed for all traits when combining scenarios.

## 4 Discussion

Water is one of the most important factors limiting crop growth. Maize requires a large amount of water throughout all stages of development, from seed germination to the reproductive phase. In this context, the significant effect of water supply levels reveals contrasting conditions in WW and WS, indicating that the irrigation treatments used in the present study to generate contrasting environments were sufficient for all traits (**Table 1**). Moreover, the significance of genotypes suggests that the panel used in this study exhibits genetic variability. Previous studies have also reported genetic diversity for the same tropical maize germplasm panel (Yassue et al., 2021; De Souza Silveira et al., 2024). Genetic variability is a fundamental factor for any breeding program.

However, the interaction effect shows that the responses were not differentiated for the genotypes across environments; they exhibit similar phenotypic responses to environmental changes. Genotype x environment is important when estimating heritability because it influences a trait’s genetic and environmental variation (Falconer and Mackay, 1996). The low effect of interaction also maximizes the accuracy (Assareh et al., 2012); high accuracy estimates indicate good experimental precision. Heritability was higher for plant height, followed by stem diameter, consistent with results from Sabiel et al. (2013), who reported moderate heritabilities for plant height and stem diameter in maize under water stress.

### 4.1 SNP in genotyping scenarios

Platforms such as SNP-Array and GBS are well-suited for genotyping hundreds to thousands of samples, each containing numerous SNP markers, in a single assay and at a significantly faster pace (Rasheed et al., 2017). This study had a balanced SNP distribution across chromosomes in the SNP-Array and GBS-B73 genotyping scenarios, perhaps attributed to using the same reference genome (**Table 2**). The inbred line B73 has been utilized as the reference genome for maize sequencing (Schnable et al., 2009), and an example of a reference genome-based pipeline is TASSEL-GBS.

In the GBS-Mock scenario, a smaller number of SNP markers was observed. In cases where a reference genome is not yet available, a simulated genome can perform SNP discovery, serving as a valid alternative, especially for minor crops (MaChado et al., 2023; Sabadin et al., 2022). Regarding the smaller number of markers observed in GBS-B73 compared to SNP-Array, this may be related to the low genomic coverage of GBS resulting in missing SNP (Wang et al., 2020). However, this issue can be partially addressed by using software employed in imputation, as missing SNP are imputed to fill in the gaps in obtaining intermediate genotype information.

### 4.2 GWAS and candidate genes

GWAS has emerged as a crucial tool, allowing for a systematic approach to identifying associations between thousands of genomic loci and complex traits. In this study, overall, more SNP were identified in association with the trait under ideal water supply conditions than under water deficit conditions in all genotyping scenarios (**Table 3**). A similar result was found by De Souza Silveira et al (2024), who identified more SNPs associated with root traits of tropical maize under ideal water supply conditions than those subjected to water scarcity. Moreover, Yassue et al (2021); Yassue et al, 2023) found more SNP associated with tropical maize traits not evaluated under inoculation by growth-promoting bacteria, such as plant height, stem diameter, and aboveground dry mass. These authors also consider that growth-related traits, such as plant height, stem diameter, and dry mass, are complex and controlled by many genes with small individual effects.

The genes found in the study have small effects (ASE), revealing the polygenic nature of the traits and controlling a relatively small portion of the genotypic variation (**Supplementary Table S1**). Complex traits in plants, such as height, diameter, and tolerance to environmental stresses, often have a multifactorial genetic basis involving the interaction of various genes and environmental factors. Thus, knowledge of the genomic regions associated with the traits of interest will provide insight into this genetic basis. Additionally, the study also detected a common marker associated with more than one trait at different water supply levels, indicating a possible pleiotropic effect. Bouchet et al. (2017) reported pleiotropy among phenology-related traits, such as plant height and leaf number, and Zhang et al. (2022) for maize productivity traits. Pleiotropic effects in GWAS studies can increase the complexity of understanding genetic and phenotypic relationships, indicating that phenotypes are more interconnected than initially thought. This complicates the interpretation of study results, as it may need to be clarified which phenotype is directly influenced by the variant and to what extent. In genetic improvement studies, pleiotropic effects can affect the selection of desirable traits, as a single genetic variant can influence multiple agronomic or desirable traits.

The candidate gene *Zm00001d005090 is* associated with SD under both water conditions and SDM under water deficit, possibly indicating a pleiotropic effect regulating the expression of these two traits. This gene is responsible for the clathrin heavy chain, one of the main subunits of clathrin, an essential protein in eukaryotic cells playing a crucial role in the endocytosis process. Hence, endocytosis occurs in many vital processes for plant development, such as abscisic acid (ABA) responses (Sutter et al., 2007). These authors state that in situations involving ABA, specific proteins in the plasma membrane are negatively regulated through the induction of their endocytosis. It has been demonstrated that ABA and salicylic acid positively regulate a gene encoding a clathrin chain in maize (Zeng et al., 2013). ABA is produced in various parts of plants, including the stem, and it influences gene expression by activating stress-response protein-coding genes and repressing growth-related genes. There is also evidence that clathrin impacts Arabidopsis’s stomatal function, gas exchange, and vegetative growth (Larson et al., 2017). Thus, this gene may have a pleiotropic effect, resulting in reduced height, stem diameter growth, and dry mass.

SNP were found to be associated with the trait simultaneously in both water availability levels, such as the gene *Zm00001d017978* identified in association with the PH trait in the SNP-Array scenario and the gene *Zm00001d001852* in association with the SD trait in the GBS-Mock scenario. *Zm00001d017978* has a putative function in the endoglucanase enzyme, a subgroup of a larger enzyme family called cellulase. Cellulases are part of a superfamily of enzymes called hydrolases that use water to break down molecules. All cellulases are essential to degrading cellulose, a structural polysaccharide found in plant cell walls (Rahman et al., 2018). The cell wall plays a crucial role in plants’ support and mechanical support, allowing them to grow by providing rigidity and resistance. Therefore, any alteration in cellulose degradation, caused by overexpression or underexpression of enzymes can affect structural integrity and, consequently, plant height. The applied water deficit may have negatively affected stem elongation, contributing to plant height, as at the V6 stage, the stem initiates the accelerated elongation phase. The gene *Zm00001d001852* has a putative function as Gibberellin-regulated protein 2 (GRP) with expression positively regulated by gibberellin. The plant hormone gibberellin regulates major aspects of plant growth and development (Yamaguchi, 2008), stimulating cell division and growth. The effect of gibberellin on stem diameter may be related to cell division and radial expansion of cells, increasing the number of cell layers. Additionally, there is evidence that biotic stresses impact gibberellin and GRP levels, as it has been reported that a slight increase in temperature can raise endogenous gibberellin concentration (Camut et al., 2019).

The genes *Zm00001d042735* and *Zm00001d031759* were also identified at both water supply levels and are associated with the SPAD trait. The first one was identified in the GBS-Mock scenario, while the other one was identified in both the SNP-Array and GBS-B73 scenarios, and both belong to the zinc finger family. Zinc finger proteins are named for their three-dimensional structure resembling a finger, binding to zinc ions through amino acids in the peptide sequence and widely distributed in eukaryotic organisms (Han et al., 2020). They bind to specific genetic sequences, interact with various proteins, participate in signal transduction, and regulate gene expression, playing an essential role in growth, development, and environmental adaptation. *Zm00001d042735* was described as a RING-type E3 ubiquitin transferase. Ubiquitin is a protein that acts as a molecular marker, signaling various cellular functions such as protein degradation, cell cycle regulation, cellular stress response, and intracellular signaling (Lee and Kim, 2011). E3 ubiquitin proteins respond to water stress by regulating ABA biosynthesis and signal transduction, modifying and degrading stress-related proteins (Han et al., 2022). An example is ZmAIRP4, which is involved in maize’s ABA signaling, and this gene’s overexpression increased water stress tolerance in *Arabidopsis* (Yang et al., 2018). Changes in water content induced by water stress can directly affect the SPAD index and chlorophyll content, as ABA concentration increases, causing stomatal closure to reduce water loss, which may affect the expression of genes related to stress response.

The gene *Zm00001d031759*, also belonging to the zinc finger protein family, has a putative function in the Protein shoot gravitropism 5 group, acting in the morphogenesis of aerial organs and responses to gravitropism. Some genes from the shoot gravitropism family have been identified and are involved in the perception and signal transduction for gravity associated with the branching angle (Yamauchi et al., 1997). It has also been found that loss of functionality of the shoot gravitropism five gene (SGR5) resulted in decreased starch accumulation in aerial tissues and consequently reduced gravity sensitivity (Tanimoto et al., 2008). Gravity is an important regulator of plant architecture, allowing plants to optimize their position relative to the soil for nutrient absorption and to light for photosynthesis. Furthermore, some genes and regions manifest for the expression of the trait independently of the water supply level, probably unrelated to water stress.

#### 4.2.1 Genes associated with phytohormone signaling pathway

Genes and regions shared among the genotyping scenarios were identified based on their function for the same trait (**Supplementary Table S3**). For example, genes *Zm00001d026477* in SNP-Array and *Zm00001d027695* in GBS-B73 are related to jasmonic acid (JA) response, associated with SPAD in WW traits. Jasmonate ZIM domain proteins, well-known as JAZ proteins, play a crucial role in pathogen responses (Ishiga et al., 2013) and are important signaling molecules in the JA pathway (Liu et al., 2017). Glutaredoxins are associated with water-induced stress response in maize, also participating in the abiotic stress response mediated by JA and ethylene through their interaction with transcription factors (Ding et al., 2019). As JA is involved in various signaling pathways regulating physiological and molecular processes in plants, in defense against biotic and abiotic stresses, such as drought (Rehman et al., 2023), signaling pathways induce stomatal closure, activating potassium efflux in guard cell protoplasts (Evans, 2003) enhancing the plants’ ability to cope with environmental stresses.

Regarding ABA regulation, *Zm00001d016786* was associated with SDM in WS in SNP-Array and *Zm00001d005090* in GBS-B73. *Zm00001d021708* was found in GBS-B73, and *Zm00001d012719* in GBS-Mock for PH under WW conditions. *Zm00001d005090* and *Zm00001d053262* were also identified in GBS-B73 and GBS-Mock, respectively, for SD under WS conditions. Protein disulfide-isomerase (PDI) is a member of the thioredoxin superfamily of redox proteins with multiple physiological functions (Khan and Mutus, 2014), playing a crucial role in abiotic stress tolerance. Thioredoxin (TRXo1) is involved in ABA perception through redox regulation of specific receptors (De Brasi-Velasco et al., 2023). In maize, genes related to PDI were highly responsive to ABA and water stress (Liu et al., 2009). A PDI-like protein strongly associated with aboveground biomass and leaf size was also identified (Kang et al., 2015). According to Tanz et al. (2012), PDI is a family of proteins that affect chlorophyll biosynthesis in Arabidopsis seedlings.

The PPR (pentatricopeptide repeat) proteins are located in mitochondria or chloroplasts. In contrast, the BZIP (basic leucine zipper) proteins constitute a family of transcription factors (TFs) associated with plant growth, development, and stress responses. A typical PPR protein is targeted to mitochondria or chloroplasts, binds to one or several organellar transcripts, and influences their expression by altering RNA sequence, turnover, processing, or translation (Barkan and Small, 2014). It has been found that the PPR96 protein, located in mitochondria, altered the transcription levels of various stress-responsive genes under ABA treatments (Liu et al., 2016b). BZIP proteins are involved in multiple stress responses, primarily through the ABA signaling pathway (Uno et al., 2000). Changes in the transcription levels of maize BZIP TFs were observed in response to ABA treatments (Cao et al., 2019).

As mentioned earlier in SDM and SD, the Clathrin heavy chain indicates possible pleiotropy. Calcium-dependent lipid-binding proteins act in response to abiotic stress, such as drought. The expression of sANN3, a calcium-dependent lipid-protein, increased in response to water stress in rice, inducing various genes in the ABA signaling pathway and promoting root growth to enhance water absorption and stomatal closure to reduce water loss (Li et al., 2019). Therefore, these proteins and the biosynthesis pathways in ABA regulation may influence photosynthesis, plant development, and growth.

The genes *Zm00001d014899* in GBS-B73 and *Zm00001d001852* in GBS-Mock are associated with the trait SD under WW conditions, involved with the phytohormone gibberellin. The first encodes a protein from the tetratricopeptide repeat (TPR)-like superfamily. Proteins containing tetratricopeptide repeats play an important role in protein-protein interaction and regulating various cellular functions (Rosado et al., 2006). They serve different crucial roles in plants, including their involvement in phytohormone signaling, such as gibberellin (Jacobsen and Olszewski, 1993; Silverstone et al., 2007). Therefore, TPR-repeat-containing proteins are pivotal in signaling phytohormones and regulating various physiological processes, including growth, development, and environmental response. Gibberellin-regulated protein 2 (GRP) was mentioned earlier, occurring at both levels of water availability for SD.

Genes associated with SDM under WW conditions were found on the same chromosome, *Zm00001d031445* in the SNP-Array and *Zm00001d027626* in the GBS-B73, both involved in ethylene biosynthesis. The ethylene-insensitive3-like/ethylene-insensitive3 (EIL/EIN3) is one of the major regulatory families in ethylene signaling, also serving as a hub for ethylene connections with various plant responses to different environmental conditions (Wu and Yang, 2019). Ethylene is a crucial regulator in stress signaling, and its interaction with a receptor complex triggers the inactivation of kinase response, resulting in the initial dephosphorylation of EIN2, followed by the cleavage of the C-terminal of EIN2. Subsequently, EIN2 translocates to the nucleus, regulating the activation of EIN3/EIL1. These proteins, in turn, exert control over ethylene response factors (Yoshida et al., 2011).

S-adenosyl-L-methionine synthetase, known as SAM, is a donor of methyl groups in the biosynthesis of nucleic acids, proteins, lipids, polysaccharides, and secondary compounds (Heidari et al., 2020). SAM is involved in many important biological processes, such as ethylene biosynthesis. Yu et al. (2012) found that alterations in the expression level of SAM affected protein synthesis, phytohormones (JA and ethylene), and genes related to stress defense response. Ethylene is a volatile compound produced endogenously by plants for growth regulation - roots, stems, leaves, and flowers (Shilev, 2020). Plants increase the synthesis of this hormone when subjected to stressful situations, whether biotic or abiotic. Water deficit, in particular, is one of the main factors related to its increase (Apelbaum and Yang, 1981). Thus, the plant alters its growth rates, decreases biomass, and reduces development (Glick, 2014).

#### 4.2.2 Genes associated with the circadian clock

*Zm00001d044194* was identified in the SNP-Array, and *Zm00001d018127* in the GBS-B73 under WW condition associated with the SPAD trait acting in the circadian clock. The MYB proteins constitute one of the most extensive families of transcription factors found in plants, playing an important role in growth and development, with widespread expression in the development of corn and soybeans in stress responses, and are closely correlated with the circadian rhythm (Du et al., 2013). MYB-related genes can act as repressors and activators associated with the circadian clock (Kamioka et al., 2016; Hsu et al., 2013; Hu et al., 2024; Schaffer et al., 1998).

The SNW/Ski domain protein is involved in the post-transcriptional regulation of circadian clock genes. SkipP interacts with the serine/arginine-rich spliceosomal protein 45 (SR45) and controls the circadian cycle through alternative splicing of circadian clock genes under biotic stress conditions (Wang et al., 2012). The circadian clock in plants refers to an internal timing system on a cycle of approximately 24 hours that regulates plants’ behavioral and physiological processes, including photosynthesis (Niwa et al., 2009). Likely, each guard cell maintains its circadian rhythm, and a clock controlling stomatal opening seems advantageous for the plant, helping prevent unnecessary water loss through transpiration (Dodd et al., 2005; Gorton et al., 1993). Thus, besides the environmental and internal factors that influence stomatal function, the circadian pattern in regulating stomatal movements is advantageous as it can enhance photosynthetic and water use efficiency.

#### 4.2.3 Genes associated with ubiquitination regulation

The genes associated with the PH trait under WS conditions were *Zm00001d053809* in SNP-Array and *Zm00001d042481* in GBS-B73, which are related to the regulation of protein ubiquitination. Culins neddylation modulates the ubiquitin ligase activity of the complex, leading to increased ubiquitination and degradation of target proteins by the proteasome (Biswas et al., 2007; Mohanty et al., 2021; Pan et al., 2004). Neddylation is the post-translational protein modification most closely related to the regulation of protein ubiquitination (Rabut and Peter, 2008).

Ubiquitin thioesterases play a fundamental role in regulating the degradation of proteins marked with ubiquitin in plants. The ubiquitin system regulates virtually all aspects of cellular function (Ernst et al., 2013), which is important in controlling abiotic stress and processes that affect agronomic traits. For example, the ubiquitin-proteasome system is an essential pathway for protein degradation in plant growth and development (Linden and Callis, 2020). The ubiquitin-proteasome system is involved in regulating transcription responsive to ABA, allowing plants to respond to abiotic stresses such as drought (Dreher and Callis, 2007). Thus, ubiquitination affects gene expression or protein abundance to determine agronomic traits and stress control, enabling dynamic adjustments in physiological and biochemical responses contributing to plant survival and adaptation under adverse conditions.

#### 4.2.4 Coincident genes among genotyping scenarios

Concerning the SNP-Array and GBS-B73 genotyping scenarios, these platforms are based on the same reference genome (B73) and are physically fixed, making it possible to determine the physical position of the marker in the genome. The coincidence between genes and regions on the same chromosome occurred only for *Zm00001d031445* in the SNP-Array and *Zm00001d027626* in the GBS-B73, both on chromosome 1. However, it was observed that, even though there was no coincidence regarding the physical position of the markers and chromosomes, there was still similarity regarding the gene functions.

Considering the three scenarios, when identifying the gene and region, it was observed that there was coincidence only for one marker in the SPAD trait under both irrigation conditions. However, possible coincidences were highlighted when deeper analyses were conducted regarding the gene function. Negro et al. (2019) concluded that GBS and SNP-Array were complementary for detecting QTLs in maize, marking different haplotypes. In a study performed in barley by Darrier et al. (2019), GBS and SNP-Array were shown to be efficient in accessing diversity. Still, they accessed different regions of the genome. These are methods that will capture different SNPs, there will be differences in position, density and distribution of the marks. However, even though they captured different regions, there was a positive correlation between the similarity matrices of both approaches. Thus, even when accessing different genome regions, these platforms demonstrate that they can be complementary. In the study, there was also a coincidence for the simulated genome, GBS-Mock, validating the complementarity for this scenario.

### 4.3 Association of markers in genotyping scenarios

The correlation between the markers in the SNP-Array and GBS-Mock scenarios provides information about the location of the markers on the chromosomes. Identifying a marker highly correlated with the GBS-Mock suggests that this marker is likely on a specific chromosome. The strength of the correlation between two markers is related to their physical proximity; the closer the markers are, the stronger the linkage disequilibrium (LD) (Myles et al., 2009). When markers are closer, they are more likely to be inherited together, leading to a stronger correlation between them. This is because when two markers are very close, they have fewer opportunities for recombination during meiosis, the process of gamete formation, which maintains stable combinations of adjacent alleles across generations. This information can be useful for guiding research and providing an initial direction for investigating the specific position of the marker in the genome.

However, according to the study results, the markers are located throughout the genome and not necessarily physically close. In other words, despite the relationship between the correlation’s strength and the markers’ physical proximity, the results showed that the markers are distributed across the entire genome. This suggests that other factors, besides physical proximity, may influence the correlation between the markers, such as genetic inheritance patterns, recombination rate, and genomic structure, highlighting the importance of considering these aspects.

### 4.4 Combining genotyping scenarios

Combining genotyping scenarios can be a valid alternative for GWAS studies, providing higher resolution results than those obtained in isolated scenarios. In the approach involving Array and GBS, it was noticed that one tool complements the other, regardless of how GBS data are explored, whether with the referenced genome or *in-silico*, as there was little difference between SNP-Array + GBS-B73 and SNP-Array + GBS-Mock. Using multiple genotyping platforms, capturing a broader range of genetic markers in linkage disequilibrium with the loci of interest is possible. This can increase the ability to detect significant associations between genetic variants and phenotypes in GWAS studies.

Concerning the use of simulated genomes, MaChado et al. (2023) and Sabadin et al. (2022), assert that it is an excellent strategy for studies on diversity, population structure, heterotic group definition, tester selection, and genomic prediction for minor crops. Another caveat is that using temperate germplasm as a reference genome may introduce a significant bias when analyzing tropical germplasm (Xu et al., 2017). As a result, favorable alleles hidden in tropical maize, in specific tropical genomic regions, may be lost (Rasheed et al., 2017). With GBS, marker discovery and genotyping occur simultaneously, mitigating this bias and enabling the identification of markers in the analyzed diversity panel (Heslot et al., 2013). Furthermore, combining information obtained via conventional approaches with a reference genome obtained from the simulated genome should improve accuracy in association studies and impact the advancement of genetic research and the development of breeding strategies.

## 5 Final remarks

Negro et al. (2019) and Darrier et al. (2019) highlighted the complementarity between standard genotyping platforms for GWAS, demonstrating that both SNP-Array and GBS can identify markers strongly linked to genes influencing key phenotypic traits. However, adopting different genotyping platforms may incur substantial costs due to their distinct methodologies. Conversely, GBS genotyping offers the flexibility to utilize both the reference genome and in-silico genome, thereby avoiding additional expenses associated with combining these scenarios. In our study, combining GBS-B73 and GBS-Mock datasets resulted in a notable increase in accuracy for several traits compared to the highest accuracy achieved by GBS alone. Specifically, we observed accuracy gains of 0.06, 0.03, 0.05, and 0.15 for SPAD, PH, SD, and SDM, respectively. This integration of datasets allows for more comprehensive analyses, capturing a broader range of SNPs and providing enhanced resolution in explaining phenotypic variation. Ultimately, leveraging a single genotyping method enables more informative and efficient data exploration, facilitating a deeper understanding of the genetic basis of traits and informing crop improvement strategies.

Indeed, when a study aims to uncover greater genetic polymorphism within a species, and SNP-Array technology is unavailable, leveraging GBS approaches becomes a viable alternative. By conducting GWAS using GBS methods, researchers can effectively identify additional polymorphisms, thereby increasing the resolution and depth of the study. This strategy is particularly beneficial for minor or orphan crops with a genome reference but need access to SNP-Array technology. In such cases, GBS offers a cost-effective and accessible means to explore the genetic diversity present within these crops, facilitating a more comprehensive understanding of their genetic architecture and potential avenues for crop improvement. By harnessing the power of GBS-based GWAS, researchers can unlock valuable insights into the genetic factors underlying traits of interest, ultimately contributing to the development of improved varieties tailored to the specific needs of these crops.

Maize, with its high genetic diversity, can target pangenomes to improve the accuracy of genetic and phenotypic analyses (Lu et al., 2015). Pangenomes offer a more comprehensive representation of genomic variations within the species, allowing for the capture of rare or subpopulation-specific variations that may not be present in a single reference genome (Marschall et al., 2018). However, if the resources needed to generate or utilize pangenomes are not available, GBS remains an effective and accessible alternative. Compared to other approaches, GBS provides greater flexibility and sufficient resolution to identify significant polymorphisms, contributing to the exploration of genetic diversity and the advancement of breeding programs. By leveraging the power of GBS-based GWAS, either alongside pangenomes or as an alternative to them, researchers can unlock valuable insights into the genetic factors underlying traits of interest. This ultimately contributes to the development of improved varieties tailored to the specific needs of these crops.

## Data Availability

The datasets presented in this study can be found in online repositories. The names of the repository/repositories and accession number(s) can be found in the article/**Supplementary Material.**

## Footnotes

1 maizegdb.org

2 bar.utoronto.ca

3 maize.uga.edu
