# Supplementary material for: Combining genotyping approaches improves resolution for association mapping: a case study in tropical maize under water stress conditions
Source: Front Plant Sci. 2025 Jan 23;15:1442008. doi: 10.3389/fpls.2024.1442008 (PMC11798985; doi:10.3389/fpls.2024.1442008)
Supplement: Supplementary file 1 [file DataSheet1.pdf]

Supplementary Material

Combining genotyping approaches improves resolution for association mapping: a case study in tropical maize under water stress conditions

Fernanda Carla Ferreira de Pontes*, Ingrid Pinheiro Machado, Maria Valnice de Souza Silveira, Antônio Lucas Aguiar Lobo, Felipe Sabadin, Roberto Fritsche-Neto, Júlio César DoVale

*** Correspondence:** Fernanda Carla Ferreira de Pontes: fernandacfpontes@alu.ufc.br

1 Supplementary Figures

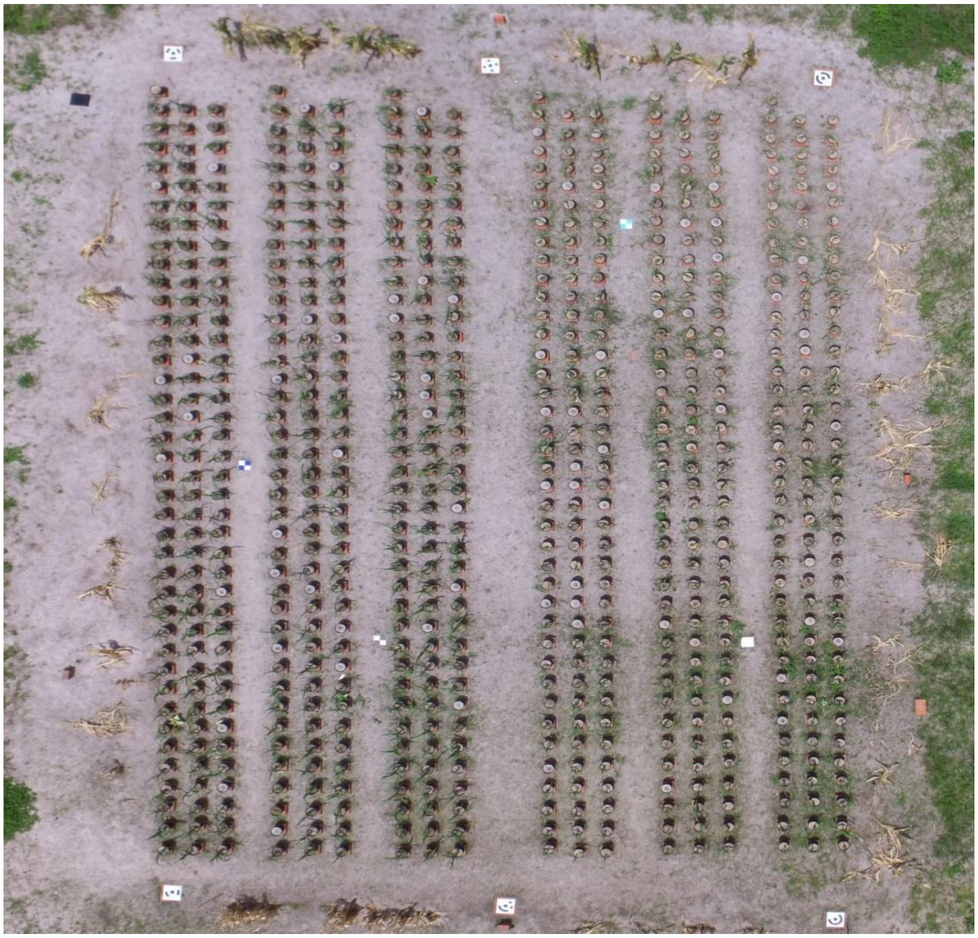

Figure S1 Aerial image showing an overview of the experimental area. The block on the right shows the WW condition and the left WS condition; the blocks are 1.5m apart.

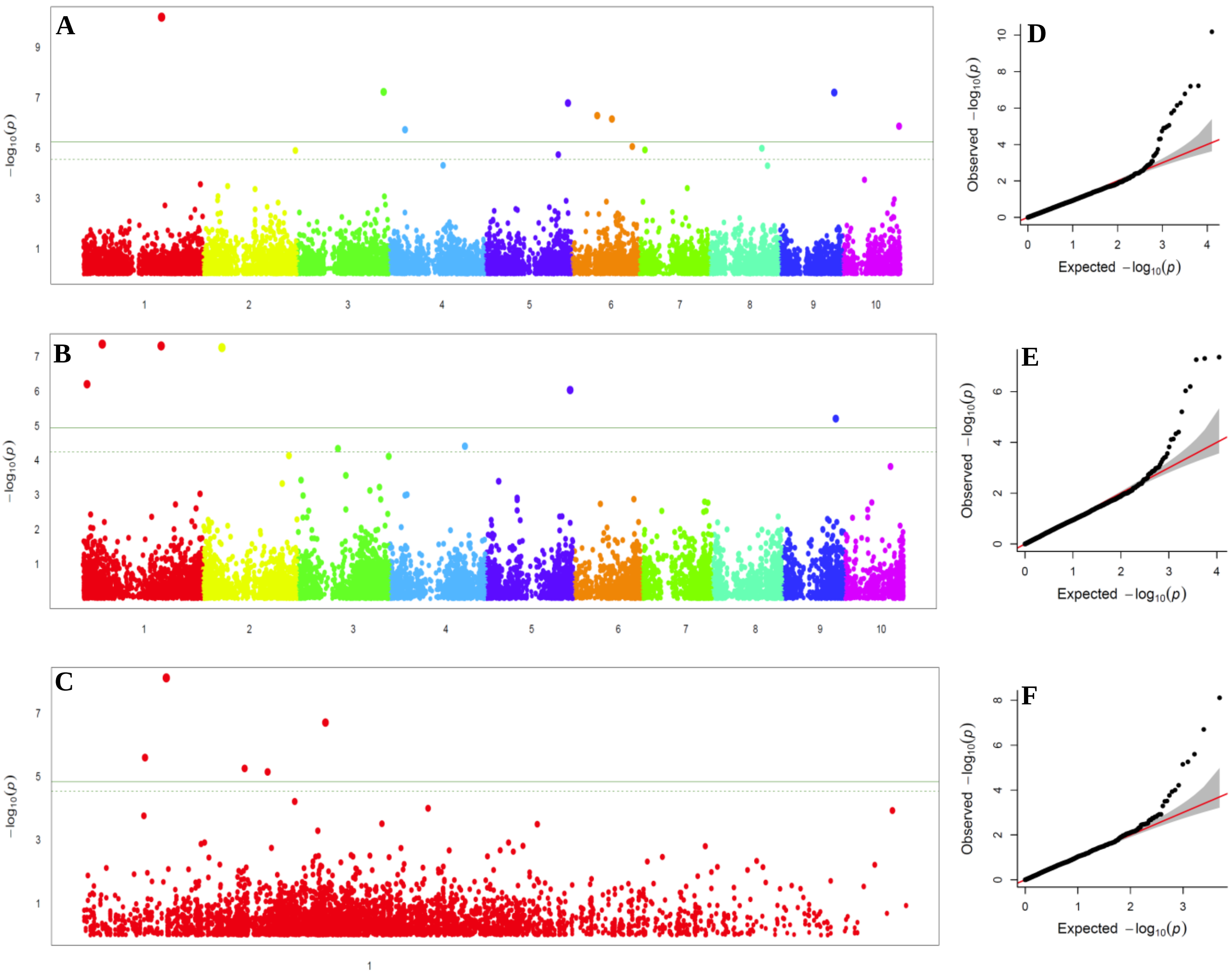

Figure S2 Manhattan plot and Quantile-Quantile (Q-Q) plots for Genome-Wide Association Study (GWAS) comparing genotyping platforms in tropical maize for SPAD trait under Well-Water (WW) conditions. The Manhattan plot displays GWAS results based on three datasets: SNP-Array **(A)**, GBS-B73 **(B)**, and Mock **(C)**. The x-axis represents the chromosomal positions, while the y-axis indicates the -log10 P-values, reflecting statistical significance. The horizontal lines denote the genome-wide suggestive significance threshold, with dots above these lines marking significant SNPs. The Q-Q plots illustrate the GWAS results for the same datasets: SNP-Array **(D)**, GBS-B73 **(E)**, and Mock **(F)**. The x-axis corresponds to the -log10 expected P-values derived from the chi-square distribution, while the y-axis represents the -log10 observed P-values. Each dot represents an SNP, with the most significant SNP appearing as the top hit. The red diagonal line shows the expected distribution under the null hypothesis of no association.

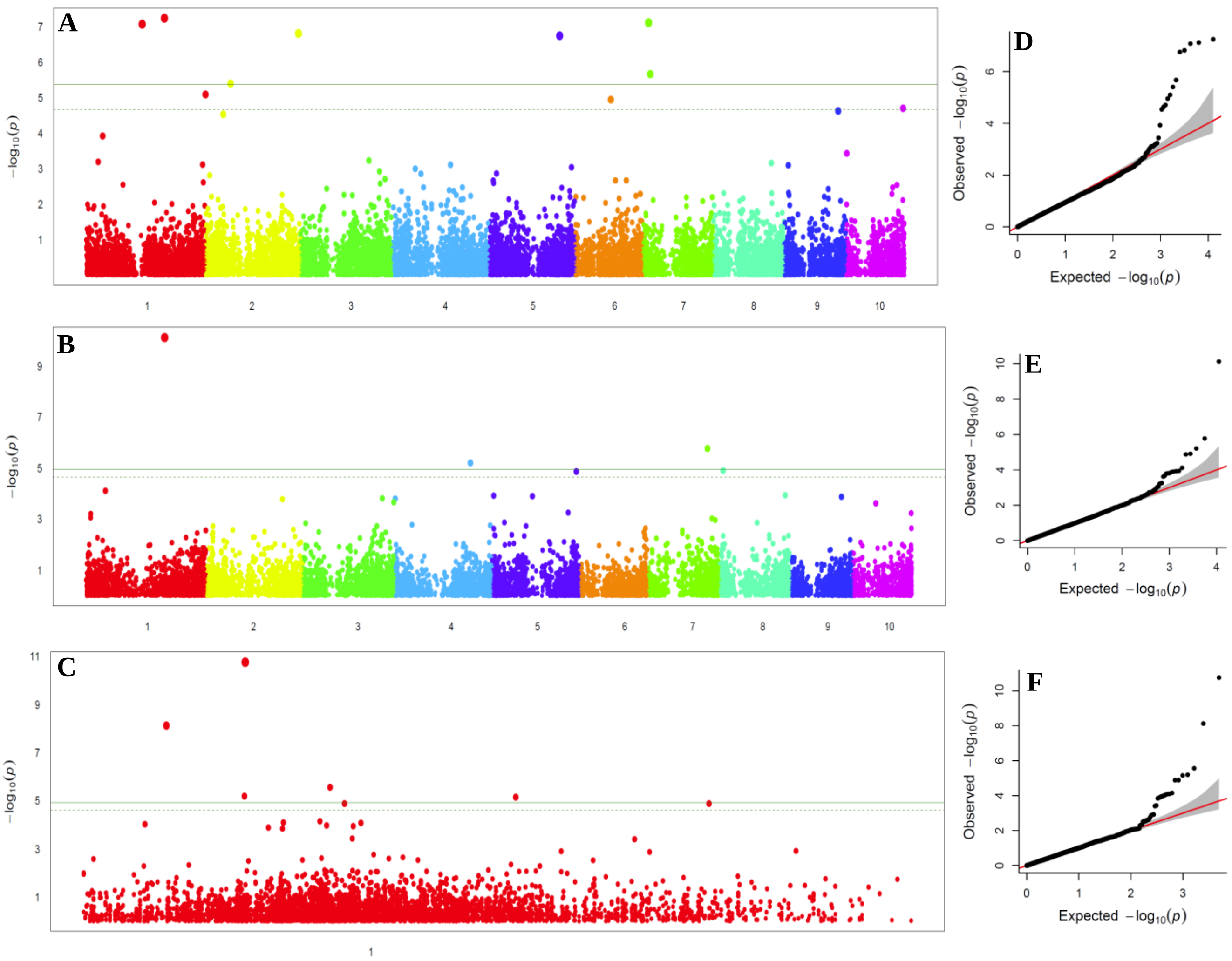

Figure S3 Manhattan plot and Quantile-Quantile (Q-Q) plots for Genome-Wide Association Study (GWAS) comparing genotyping platforms in tropical maize for SPAD trait under Water-Stressed (WS) conditions. The Manhattan plot displays GWAS results based on three datasets: SNP-Array **(A)**, GBS-B73 **(B)**, and Mock **(C)**. The x-axis represents the chromosomal positions, while the y-axis indicates the -log10 P-values, reflecting statistical significance. The horizontal lines denote the genome-wide suggestive significance threshold, with dots above these lines marking significant SNPs. The Q-Q plots illustrate the GWAS results for the same datasets: SNP-Array **(D)**, GBS-B73 **(E)**, and Mock **(F)**. The x-axis corresponds to the -log10 expected P-values derived from the chi-square distribution, while the y-axis represents the -log10 observed P-values. Each dot represents an SNP, with the most significant SNP appearing as the top hit. The red diagonal line shows the expected distribution under the null hypothesis of no association.

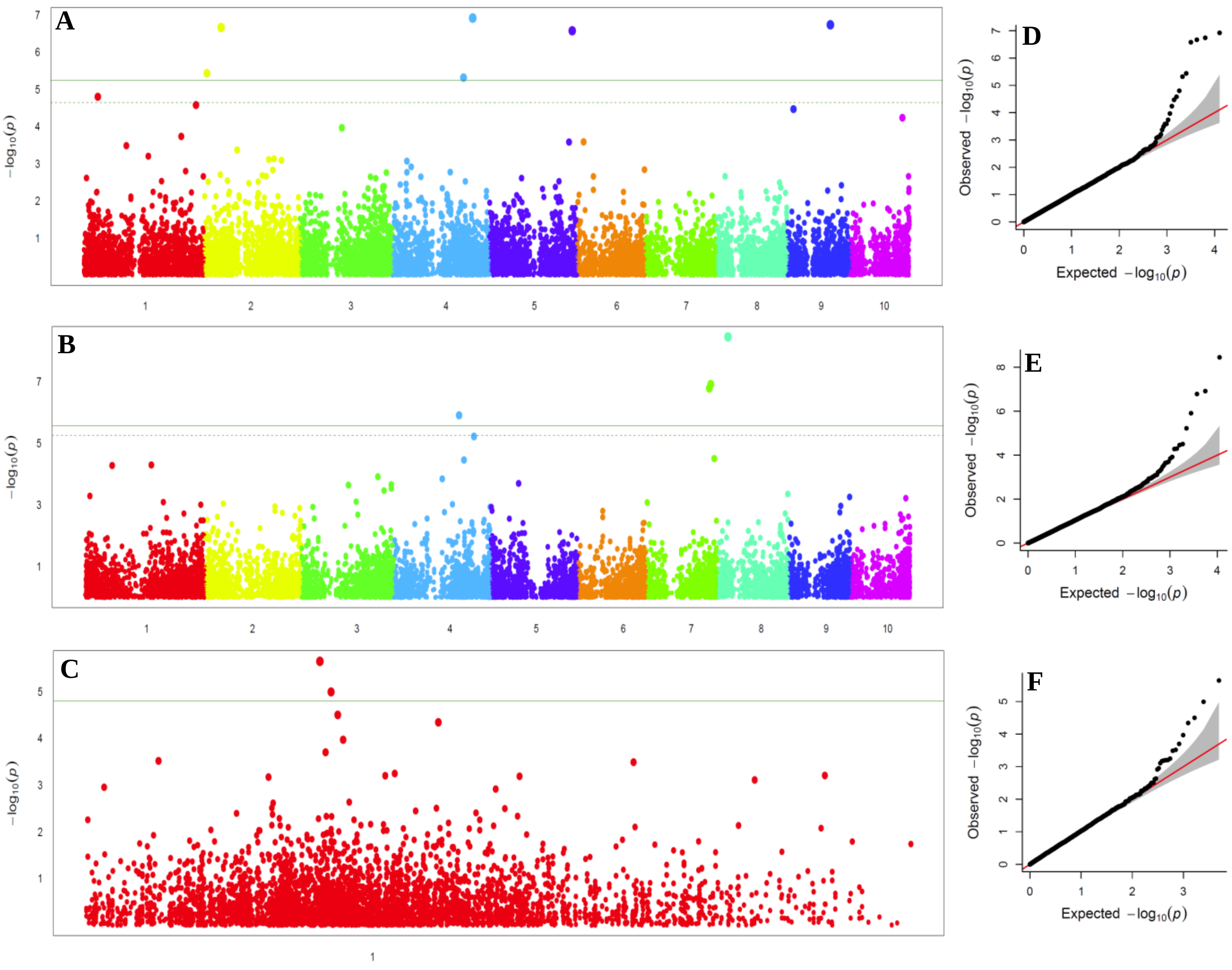

Figure S4 Manhattan plot and Quantile-Quantile (Q-Q) plots for Genome-Wide Association Study (GWAS) comparing genotyping platforms in tropical maize for plant height (PH) trait under Well-Watered (WW) conditions. The Manhattan plot displays GWAS results based on three datasets: SNP-Array **(A)**, GBS-B73 **(B)**, and Mock **(C)**. The x-axis represents the chromosomal positions, while the y-axis indicates the -log10 P-values, reflecting statistical significance. The horizontal lines denote the genome-wide suggestive significance threshold, with dots above these lines marking significant SNPs. The Q-Q plots illustrate the GWAS results for the same datasets: SNP-Array **(D)**, GBS-B73 **(E)**, and Mock **(F)**. The x-axis corresponds to the -log10 expected P-values derived from the chi-square distribution, while the y-axis represents the -log10 observed P-values. Each dot represents an SNP, with the most significant SNP appearing as the top hit. The red diagonal line shows the expected distribution under the null hypothesis of no association.

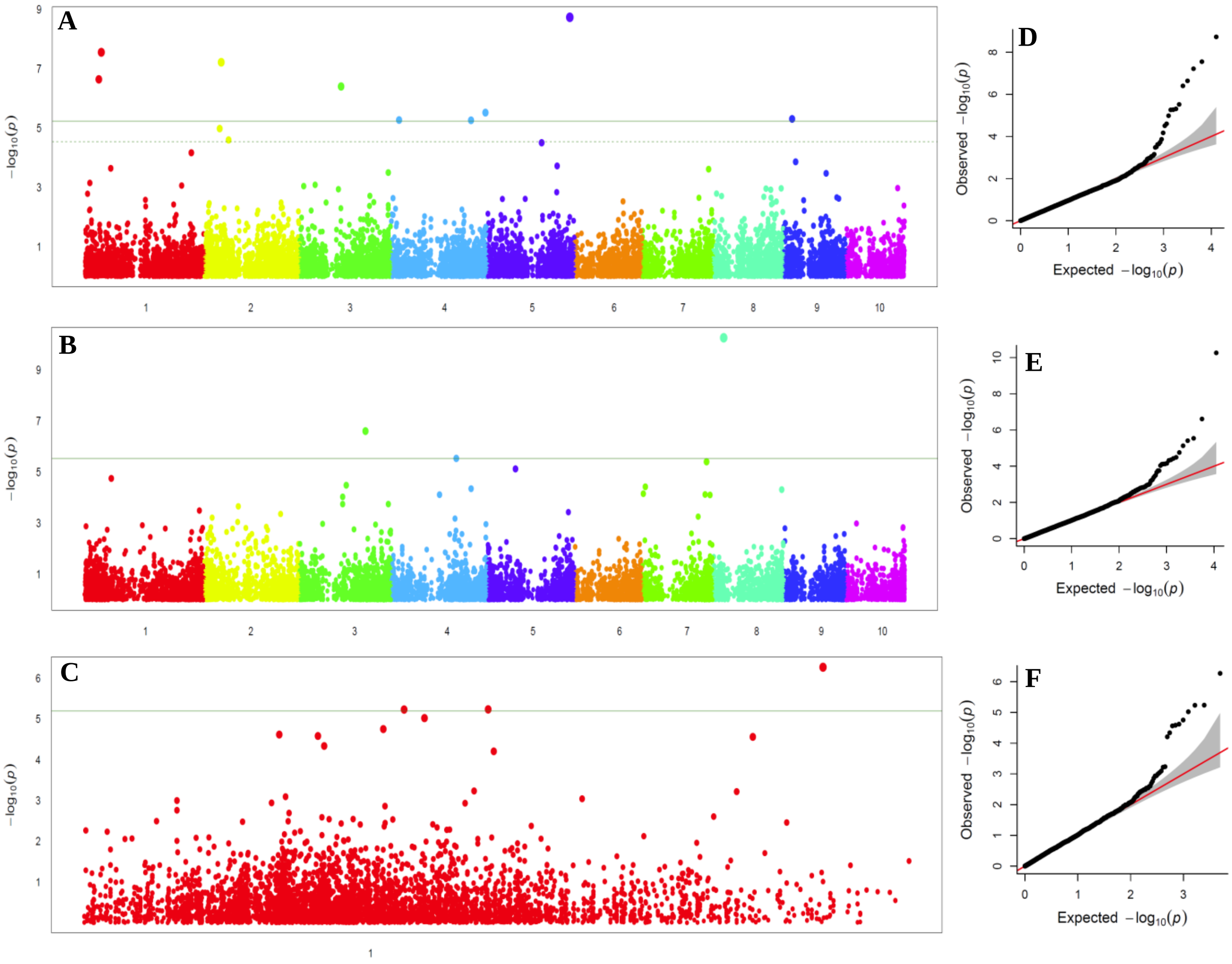

Figure S5 Manhattan plot and Quantile-Quantile (Q-Q) plots for Genome-Wide Association Study (GWAS) comparing genotyping platforms in tropical maize for plant height (PH) trait under Water-Stressed (WS) conditions. The Manhattan plot displays GWAS results based on three datasets: SNP-Array **(A)**, GBS-B73 **(B)**, and Mock **(C)**. The x-axis represents the chromosomal positions, while the y-axis indicates the -log10 P-values, reflecting statistical significance. The horizontal lines denote the genome-wide suggestive significance threshold, with dots above these lines marking significant SNPs. The Q-Q plots illustrate the GWAS results for the same datasets: SNP-Array **(D)**, GBS-B73 **(E)**, and Mock **(F)**. The x-axis corresponds to the -log10 expected P-values derived from the chi-square distribution, while the y-axis represents the -log10 observed P-values. Each dot represents an SNP, with the most significant SNP appearing as the top hit. The red diagonal line shows the expected distribution under the null hypothesis of no association.

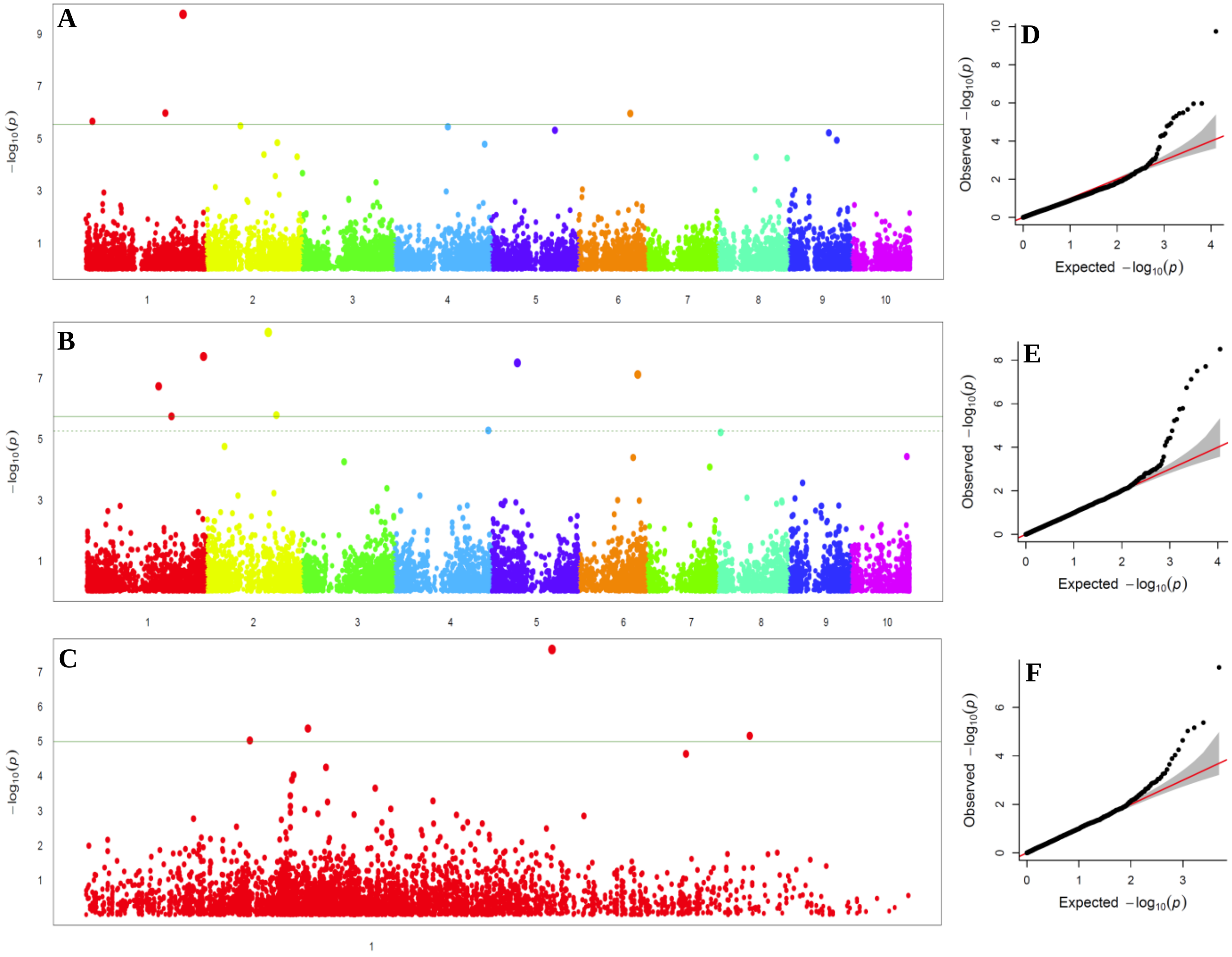

Figure S6 Manhattan plot and Quantile-Quantile (Q-Q) plots for Genome-Wide Association Study (GWAS) comparing genotyping platforms in tropical maize for stalk diameter (SD) trait under Well-Watered (WW) conditions. The Manhattan plot displays GWAS results based on three datasets: SNP-Array **(A)**, GBS-B73 **(B)**, and Mock **(C)**. The x-axis represents the chromosomal positions, while the y-axis indicates the -log10 P-values, reflecting statistical significance. The horizontal lines denote the genome-wide suggestive significance threshold, with dots above these lines marking significant SNPs. The Q-Q plots illustrate the GWAS results for the same datasets: SNP-Array **(D)**, GBS-B73 **(E)**, and Mock **(F)**. The x-axis corresponds to the -log10 expected P-values derived from the chi-square distribution, while the y-axis represents the -log10 observed P-values. Each dot represents an SNP, with the most significant SNP appearing as the top hit. The red diagonal line shows the expected distribution under the null hypothesis of no association.

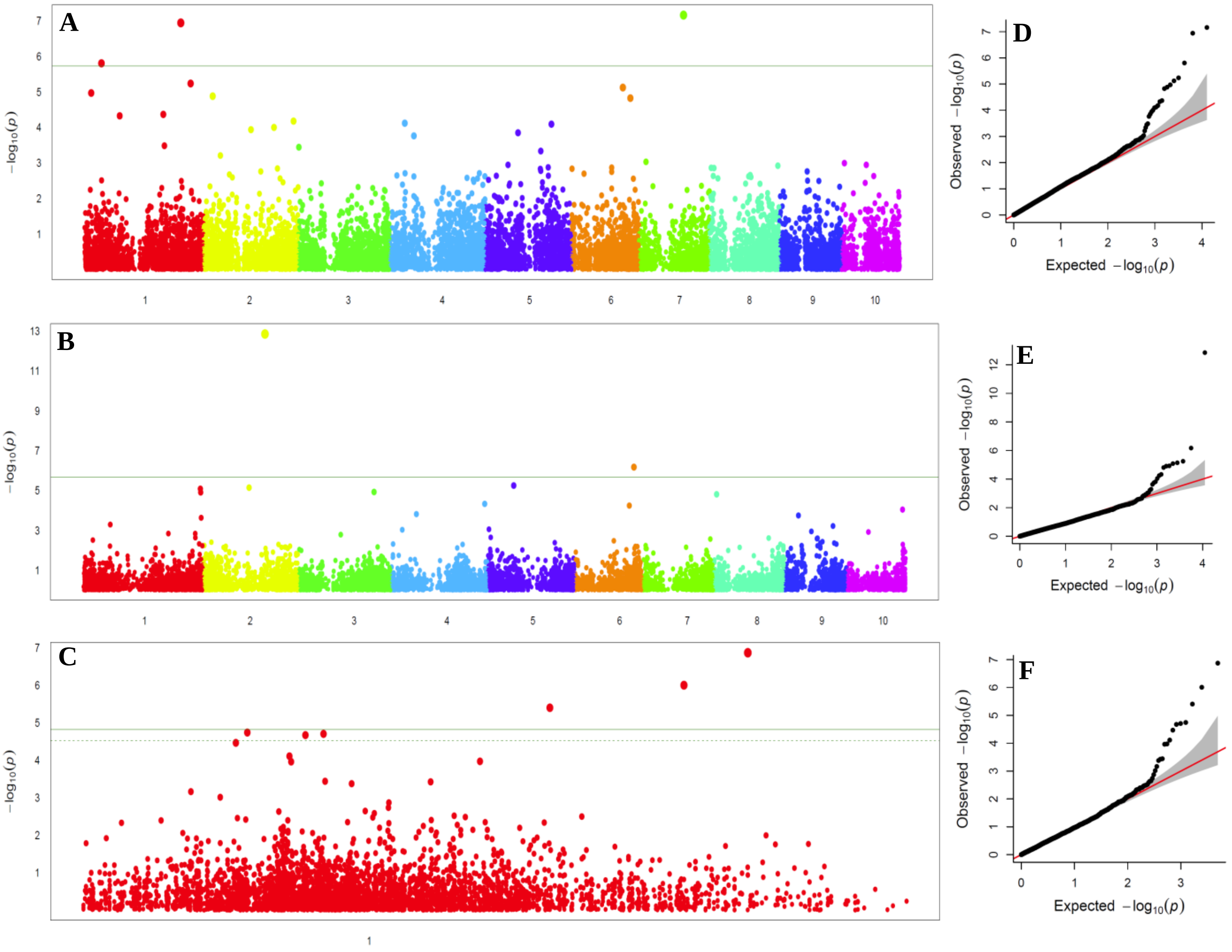

Figure S7 Manhattan plot and Quantile-Quantile (Q-Q) plots for Genome-Wide Association Study (GWAS) comparing genotyping platforms in tropical maize for stalk diameter (SD) trait under Water-Stressed (WS) conditions. The Manhattan plot displays GWAS results based on three datasets: SNP-Array **(A)**, GBS-B73 **(B)**, and Mock **(C)**. The x-axis represents the chromosomal positions, while the y-axis indicates the -log10 P-values, reflecting statistical significance. The horizontal lines denote the genome-wide suggestive significance threshold, with dots above these lines marking significant SNPs. The Q-Q plots illustrate the GWAS results for the same datasets: SNP-Array **(D)**, GBS-B73 **(E)**, and Mock **(F)**. The x-axis corresponds to the -log10 expected P-values derived from the chi-square distribution, while the y-axis represents the -log10 observed P-values. Each dot represents an SNP, with the most significant SNP appearing as the top hit. The red diagonal line shows the expected distribution under the null hypothesis of no association.

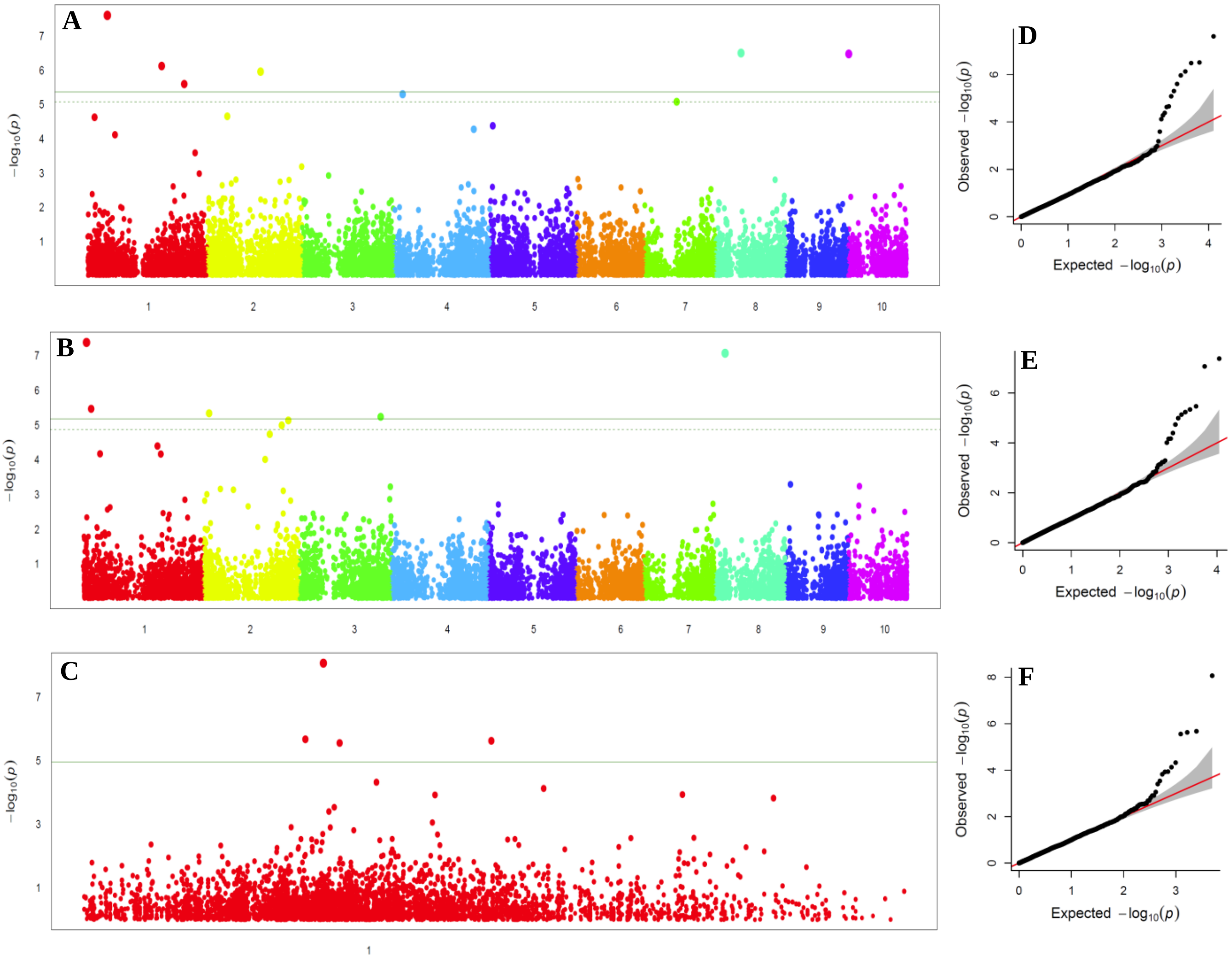

Figure S8 Manhattan plot and Quantile-Quantile (Q-Q) plots for Genome-Wide Association Study (GWAS) comparing genotyping platforms in tropical maize for shoot dry matter (SDM) trait under Well-Watered (WW) conditions. The Manhattan plot displays GWAS results based on three datasets: SNP-Array **(A)**, GBS-B73 **(B)**, and Mock **(C)**. The x-axis represents the chromosomal positions, while the y-axis indicates the -log10 P-values, reflecting statistical significance. The horizontal lines denote the genome-wide suggestive significance threshold, with dots above these lines marking significant SNPs. The Q-Q plots illustrate the GWAS results for the same datasets: SNP-Array **(D)**, GBS-B73 **(E)**, and Mock **(F)**. The x-axis corresponds to the -log10 expected P-values derived from the chi-square distribution, while the y-axis represents the -log10 observed P-values. Each dot represents an SNP, with the most significant SNP appearing as the top hit. The red diagonal line shows the expected distribution under the null hypothesis of no association.

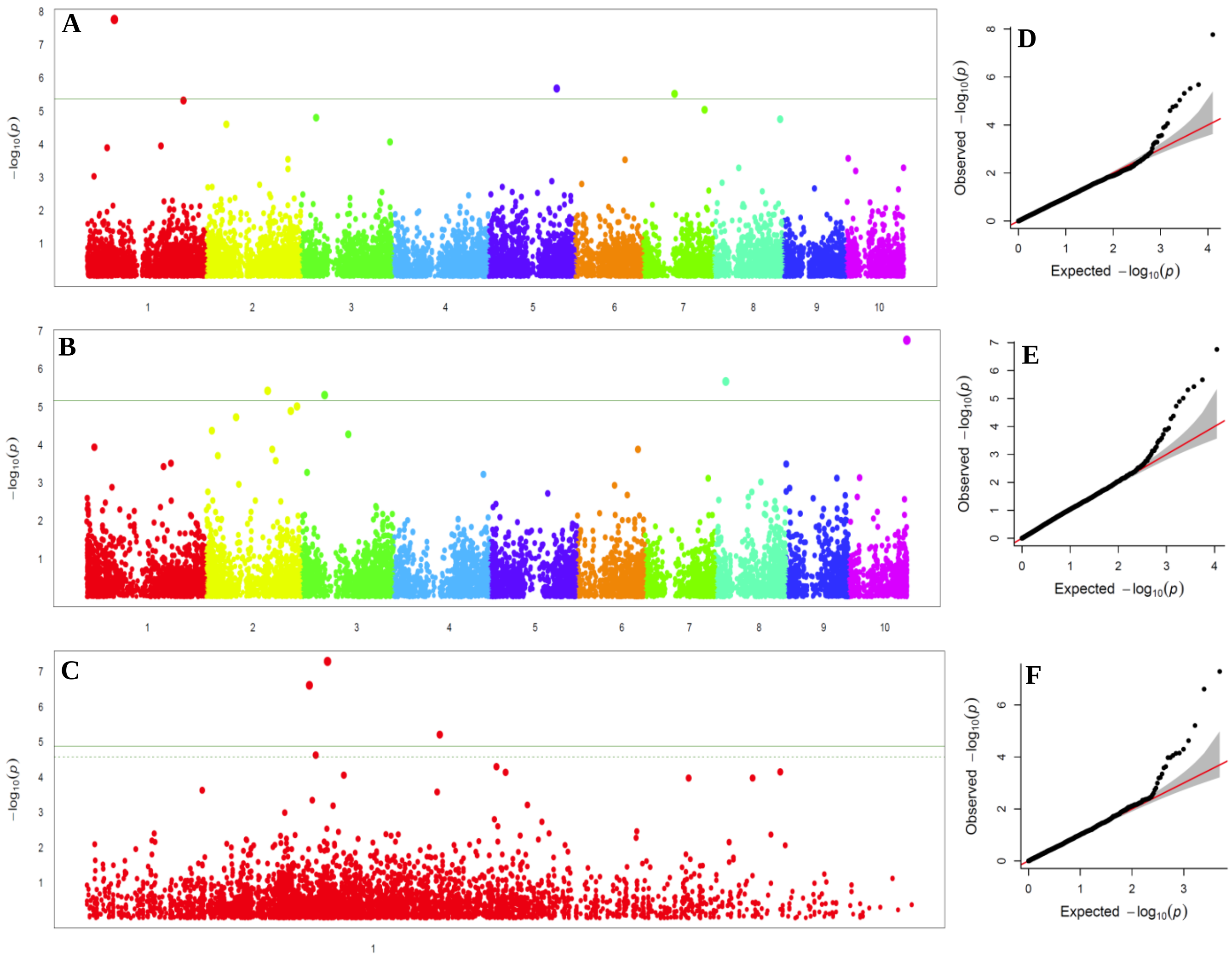

Figure S9 Manhattan plot and Quantile-Quantile (Q-Q) plots for Genome-Wide Association Study (GWAS) comparing genotyping platforms in tropical maize for shoot dry matter (SDM) trait under Water-Stressed (WS) conditions. The Manhattan plot displays GWAS results based on three datasets: SNP-Array **(A)**, GBS-B73 **(B)**, and Mock **(C)**. The x-axis represents the chromosomal positions, while the y-axis indicates the -log10 P-values, reflecting statistical significance. The horizontal lines denote the genome-wide suggestive significance threshold, with dots above these lines marking significant SNPs. The Q-Q plots illustrate the GWAS results for the same datasets: SNP-Array **(D)**, GBS-B73 **(E)**, and Mock **(F)**. The x-axis corresponds to the -log10 expected P-values derived from the chi-square distribution, while the y-axis represents the -log10 observed P-values. Each dot represents an SNP, with the most significant SNP appearing as the top hit. The red diagonal line shows the expected distribution under the null hypothesis of no association.

2 Supplementary Tables

Table S1. Marker, chromosome (Chr), physical position (pos), -log$_{10}$ of the p-value, minor allele frequency (MAF), allele substitution effect (ASE), the proportion of phenotypic variance explained by the SNP (R^2_{SNP}), and annotation of candidate genes detected by GWAS analysis for traits in three genotyping scenarios in WW (well-watered) and WS (water-stressed) conditions water supply

Trait	Scenario	Marker	Chrm	Posi (bp)	p-value	MAF	ASE	R^2_{SNP}	Putative annotation
SPAD in WW	SNP-Array	*Zm00001d031759*	1	200951693	10.19	0.33	-0.101	9e-04	Protein shoot gravitropism 5
		Zm00001d044194	3	221628210	7.22	0.47	0.067	1e-04	MYB-related-transcription factor 97
		Zm00001d049717	4	41320760	5.72	0.39	0.061	1e-04	Loricrin-related
		Zm00001d018076	5	213811740	6.78	0.30	0.076	1e-04	FLZ-type domain-containing protein
		Zm00001d036010	6	65518917	6.28	0.15	0.100	4e-04	Oligopeptide transporter 7
		Zm00001d036833	6	103276663	6.15	0.31	0.071	1e-04	Putative pentatricopeptide repeat-containing protein
		Zm00001d047696	9	139410201	7.20	0.07	-0.136	1e-04	Senescence associated gene 20
		Zm00001d026477	10	146706556	5.86	0.29	0.069	1e-04	Jasmonate ZIM-domain proten
	GBS-B73	*Zm00001d027695*	1	11300621	6.20	0.08	0.119	1e-04	Glutaredoxin-C13
		Zm00001d028901	1	50207452	7.36	0.11	0.145	9e-04	High chlorophyll fluorescence3
		Zm00001d031759	1	200951623	7.31	0.31	-0.090	4e-04	Protein shoot gravitropism 5
		Zm00001d003603	2	49593983	7.26	0.38	0.082	4e-04	Major facilitator superfamily protein
		Zm00001d018127	5	214805832	6.03	0.05	0.204	9e-04	SNW/SKI-interacting protein
		Zm00001d047574	9	136093835	5.21	0.36	-0.068	1e-04	WRKY domain-containing protein
	GBS-Mock	*Zm00001d036175*	Unique	76554426	5.60	0.09	0.114	1e-04	GDSL esterase/lipase APG
		No hits found	Unique	103179336	8.11	0.15	0.123	9e-04	none
		Zm00001d042735	Unique	201318938	5.26	0.06	0.181	9e-04	RING-type E3 ubiquitin transferase
		Zm00001d042755	Unique	230114762	5.15	0.05	0.137	1e-04	none
		Zm00001d024497	Unique	302570660	6.70	0.38	-0.081	4e-04	none
SPAD in WS	SNP-Array	*Zm00001d030556*	1	143751621	7.08	0.36	0.073	1e-04	Organic cation/carnitine transporter 7
		Zm00001d031759	1	200951693	7.25	0.33	-0.082	4e-04	Protein shoot gravitropism 5
		Zm00001d003871	2	64226257	5.40	0.40	0.061	1e-04	AP2-EREBP-transcription factor 91
		Zm00001d007742	2	238555551	6.82	0.07	-0.130	1e-04	none
		Zm00001d017043	5	183519054	6.75	0.10	-0.113	1e-04	OSJNBb0022F23.8-like protein
		Zm00001d019062	7	14881267	7.12	0.24	-0.084	4e-04	membrane H(+)-ATPase3
		Zm00001d019150	7	19277460	5.67	0.09	-0.112	1e-04	Pathogenesis-related thaumatin superfamily protein

	GBS-B73	*Zm00001d031759*	1	200951623	10.12	0.31	-0.106	2e-03	Protein shoot gravitropism 5
		Zm00001d052519	4	191970203	5.21	0.07	0.155	4e-04	Putative 2-carboxy-D-arabinitol-1-phosphatase
		Zm00001d021440	7	152284943	5.77	0.38	0.073	4e-04	HMA domain-containing protein
	GBS-Mock	*No hits found*	Unique	103179336	8.13	0.15	0.15	9e-04	none
		Zm00001d008500	Unique	200328622	5.20	0.07	0.07	1e-04	Histidine-rich calcium-binding protein
		Zm00001d042735	Unique	201318938	10.76	0.06	0.06	5e-03	RING-type E3 ubiquitin transferase
		Zm00001d006357	Unique	306796326	5.56	0.30	0.30	1e-04	Protein GRIP
		Zm00001d029023	Unique	537851163	5.15	0.23	0.23	4e-04	Hexosyltransferase
**PH in WW**	SNP-Array	*Zm00001d002163*	2	6961288	5.44	0.31	-0.077	4e-04	Diacylglycerol kinase
		Zm00001eb080540	2	43019177	6.67	0.42	-0.081	4e-04	Floury endosperm1
		No hits found	4	181112611	5.32	0.27	-0.078	1e-04	none
		Zm00001d052912	4	204485263	6.92	0.42	0.082	4e-04	Serine/threonine receptor-like kinase NFP
		Zm00001d017978	5	211170831	6.58	0.35	0.086	4e-04	Endoglucanase
		Zm00001d046890	9	109050043	6.74	0.29	0.091	4e-04	Putative ubiquitin-conjugating enzyme family
	GBS-B73	*Zm00001d051672*	4	166302733	5.91	0.11	0.105	1e-04	GPI-anchored protein
		Zm00001d021708	7	160911849	6.78	0.06	0.179	4e-04	Pentatricopeptide repeat-containing protein chloroplastic
		Zm00001d021859	7	164642302	6.91	0.45	-0.089	4e-04	ATP-dependent DNA helicase
		Zm00001d008952	8	26903022	8.45	0.29	-0.102	4e-04	Endoglucanase
	GBS-Mock	*No hits found*	Unique	291972610	5.64	0.26	0.089	4e-04	none
		Zm00001d012719	Unique	305928723	4.99	0.12	-0.108	1e-04	BZIP-transcription factor
**PH in WS**	SNP-Array	*Zm00001d028464*	1	35732896	6.64	0.08	0.143	4e-04	Rho GTPase activation protein with PH domain
		Zm00001d028660	1	42126116	7.55	0.08	0.149	4e-04	Acyl-CoA N- with type zinc finger domain
		Zm00001eb080540	2	43019177	7.21	0.42	-0.086	4e-04	Floury endosperm1
		No hits found	3	106448106	6.40	0.39	-0.078	4e-04	none
		Zm00001d049192	4	19864256	5.27	0.16	0.080	1e-04	Chaperone protein dnaJ-related
		Zm00001d052912	4	204485263	5.26	0.42	0.066	1e-04	Serine/threonine receptor-like kinase NFP
		Zm00001d053809	4	241454721	5.52	0.43	0.067	1e-04	Defective in cullin neddylation protein
		Zm00001d017978	5	211170831	8.73	0.35	0.097	4e-04	Endoglucanase
		Zm00001d045427	9	21787790	5.30	0.46	0.066	1e-04	Barren stalk fastigiate 1
	GBS-B73	*Zm00001d042481*	3	169056323	6.61	0.42	-0.078	4e-04	Ubiquitin thioesterase OTU
		Zm00001d051672	4	166302733	5.54	0.11	0.103	1e-04	GPI-anchored protein
		Zm00001d008954	8	26903022	10.26	0.29	-0.117	2e-03	Ectonucleotide pyrophosphatase
	GBS-Mock	*Zm00001d034057*	Unique	399525514	5.23	0.34	0.079	4e-04	Protein shoot gravitropism 5
		No hits found	Unique	504442276	5.24	0.36	-0.153	5e-03	none

		Zm00001d018703	Unique	922415122	6.27	0.09	0.146	4e-04	F-box domain-containing protein
**SD in WW**	SNP-Array	*Zm00001d027902*	1	17165877	5.66	0.32	-0.059	1e-04	Protein TIFY
		Zm00001d031832	1	203133117	5.98	0.46	0.059	4e-04	Ubiquitin carboxyl-terminal hydrolase 17
		Zm00001d033038	1	248268614	9.75	0.30	-0.092	3e-03	Stress enhanced protein 1 chloroplastic
		Zm00001d037612	6	131758145	5.96	0.37	-0.061	4e-04	Myb_DNA-bind_3 domain-containing protein
	GBS-B73	*Zm00001d031287*	1	185122781	6.74	0.46	-0.060	4e-04	Oligopeptide transporter 5
		Zm00001d032225	1	217881804	5.75	0.11	-0.095	4e-04	Formin-like protein
		Zm00001d034681	1	299645762	7.71	0.13	-0.106	9e-04	Pentatricopeptide repeat-containing protein
		Zm00001d005090	2	157770180	8.51	0.33	0.077	9e-04	Clathrin heavy chain
		Zm00001d005558	2	178434079	5.79	0.37	-0.055	1e-04	leucine--tRNA ligase
		Zm00001d014899	5	67342178	7.50	0.09	-0.112	4e-04	Tetratricopeptide repeat (TPR)-like superfamily protein
		Zm00001d038199	6	151389473	7.12	0.23	0.073	4e-04	MLO-like protein
	GBS-Mock	*Zm00001d026300*	Unique	205301556	5.03	0.05	-0.109	1e-04	Argonaute 2-like
		No hits found	Unique	277995367	5.37	0.22	0.097	9e-04	none
		Zm00001d001852	Unique	583680097	7.65	0.34	0.066	4e-04	Gibberellin-regulated protein 2
		Zm00001d047956	Unique	831219654	5.16	0.14	0.070	1e-04	Helicase-like transcription factor CHR27
**SD in WS**	SNP-Array	*Zm00001d028699*	1	43578111	5.80	0.38	-0.052	1e-04	Ypt/Rab-GAP domain of gyp1p superfamily protein
		Zm00001d033038	1	248268614	6.94	0.30	-0.076	4e-04	Stress enhanced protein 1 chloroplastic
		Zm00001d020444	7	114906347	7.16	0.18	-0.092	4e-04	Putative zinc finger motif protein
	GBS-B73	*Zm00001d005090*	2	157770180	12.85	0.33	0.095	2e-03	Clathrin heavy chain
		Zm00001d038199	6	151389473	6.17	0.23	0.065	1e-04	MLO-like protein
	GBS-Mock	*Zm00001d001852*	Unique	583680097	5.41	0.34	0.057	1e-04	Gibberellin-regulated protein 2
		Zm00001d053262	Unique	751364673	6.01	0.48	0.116	6e-03	Calcium-dependent lipid-binding family protein
		Zm00001d047956	Unique	831219654	6.87	0.14	0.089	4e-04	Helicase-like transcription factor CHR27
**SDM in WW**	SNP-Array	*Zm00001d028912*	1	50880303	7.61	0.07	0.138	9e-04	UPF0481 protein
		Zm00001d031445	1	190284851	6.13	0.35	0.060	9e-04	Ethylene insensitive 3-like 3 protein
		Zm00001d033038	1	248268614	5.60	0.30	-0.062	9e-04	Stress enhanced protein 1 chloroplastic
		No hits found	2	137425632	5.96	0.37	0.057	9e-04	none
		Zm00001d009468	8	65780194	6.51	0.24	-0.067	9e-04	Ereb49 - AP2-EREBP-transcription factor 49
		Zm00001d023272	10	1811784	6.48	0.29	0.059	1e-04	ENTH/VHS family protein
	GBS-B73	*Zm00001d027626*	1	9405820	7.38	0.06	0.120	9e-04	S-adenosyl-L-methionine-dependent methyltransferase superfamily protein
		Zm00001d028039	1	21295780	5.47	0.21	-0.060	1e-04	S-acyltransferase
		Zm00001d002541	2	14991268	5.34	0.14	0.070	1e-04	RING-type E3 ubiquitin transferase

		Zm00001d043706	3	207925584	5.24	0.26	0.059	1e-04	Transcription factor
		Zm00001d008944	8	26440815	7.07	0.48	0.057	9e-04	Uncharacterized protein
	GBS-Mock	*No hits found*	Unique	277995367	5.67	0.22	0.091	2e-03	none
		No hits found	Unique	300613355	8.07	0.31	-0.105	5e-03	none
		Zm00001d046354	Unique	320987424	5.56	0.19	0.068	1e-04	GATA transcription factor 20
		Zm00001d008954	Unique	511458983	5.63	0.28	-0.064	9e-04	Ectonucleotide pyrophosphatase/phosphodiesterase family member 3
**SDM in WS**	SNP-Array	*Zm00001d029438*	1	70502769	7.77	0.41	-0.066	9e-04	RING-type E3 ubiquitin transferase
		Zm00001d016786	5	175971996	5.68	0.17	-0.076	9e-04	PDIL5-3-Zea mays protein disulfide isomerase or PDI-like 5-2
		No hits found	7	82368315	5.52	0.19	0.061	1e-04	none
	GBS-B73	*Zm00001d005090*	2	157770180	5.43	0.33	0.051	1e-04	Clathrin heavy chain
		Zm00001d040705	3	59361883	5.31	0.22	-0.052	1e-04	Peroxidase
		Zm00001d008944	8	26440815	5.67	0.48	0.050	1e-04	Uncharacterized protein
		Zm00001d026638	10	149275033	6.76	0.09	-0.094	1e-04	Calcium-binding EF hand family protein
	GBS-Mock	*No hits found*	Unique	277995367	6.61	0.22	0.100	2e-03	none
		No hits found	Unique	300613355	7.29	0.31	-0.096	3e-03	none
		Zm00001d018001	Unique	440728659	5.21	0.13	0.063	1e-04	Xanthine/uracil permease family protein

Table S2. DNA fragments were obtained via BLAST in GBS-Mock for each trait in WW (well-watered) and WS (water-stressed) in water supply conditions. SPAD, PH (plant height), SD (stalk diameter), and SDM (shoot dry matter)

SPAD in WW	
**DNA fragments**	GCAGATCTGCTCACATGTTTCCGGCTCCATCGAAACAGAAC<br>TGAAGCATCCAGCTTCACATACTAGTAGACCACCTTATGGA<br>GAGTGCAATAAGATCGAAGGTCACGTCCAAAAAAAAAAAAA<br>ACGAGGTCTTAGTTCCAGAAGACAGATTCAAAAAAAAAAAA<br>TTCCCATTTCCCTCCTTCCTACAAACTGCTTCACAAAAAAA

SPAD in WS	
**DNA fragments**	TGAAGCATCCAGCTTCACATACTAGTAGACCACCTTATGGA<br>CATGGCCTGCAGCTAGACAAAGTCCGGCACCAACGAACGGC<br>GAGTGCAATAAGATCGAAGGTCACGTCCAAAAAAAAAAAAA<br>ACTCCCTGCTGCAGCAAAAAATGGTTGTTTGTAATAGTCAC<br>CTGCCTGGCGTGCGTGAGGAGTCTGGCGTCTGACAGATCGG

PH in WW	
**DNA fragments**	TCCCAGGAAAAGAAAAAAAAAAACTAAATTACAGATCGAAGA<br>CTGCAGCCCACGAGCGAGGTGGTGGAGGAGGACGCCCTGCT

PH in WS	
**DNA fragments**	CTTCGCCTTCTGCAGGTCGCGCGCACCATCCTAGAAGACAA<br>ACAAGTAGTTGTGAAGTTACCACAGCATTCATTGCTATTTG<br>ACCTGCAACACGCGAATGACCGTACCTGCTGTTCCATTGTT

SD in WW	
**DNA fragments**	AGCAATCCTGTAGAACAGACGGCAGAGAGCTGCATAACGTC<br>CGTCCAGCACGAATAGCCTTGTCCATGGCTTTATCGCGTTG<br>TAGAGCTGGACCGGCCGGGAGGGTTTTTACAGATCGGAAAA<br>AAAAAAAAAAAAAAACCTAAGAAGAAGGCCAGAGTGAGGTA

SD in WS	
**DNA fragments**	AGAGCTGGACCGGCCGGGAGGGTTTTTACAGATCGGAAAA<br>CATTGAACAGGATGGTCCAAACCCCTCTTCGCCAAACGAT<br>AAAAAAAAAAAAAACCTAAGAAGAAGGCCAGAGTGAGGTA

SDM in WW	
**DNA fragments**	CGTCCAGCACGAATAGCCTTGTCCATGGCTTTATCGCGTTG<br>TTGTTGCGCGCAATTACTTTGGATGAGGCAAACCCTTAGGG<br>CCCTTGTGGAGGAGTGGTCCATGCGGCCCCAAAGGTATCCT<br>CAGCTGTTTGGGCTGCAGCAAGGGGCGGACCATGTGTGTGC

SDM in WS	
**DNA fragments**	CGTCCAGCACGAATAGCCTTGTCCATGGCTTTATCGCGTTG<br>TTGTTGCGCGCAATTACTTTGGATGAGGCAAACCCTTAGGG<br>GTGCAAGAGCAAATACAATAATTGGCATATAGCCAAACAAA

Table S3. Marker, chromosome (Chr), physical position (pos), annotation of candidate genes, and common function detected by GWAS analysis for traits in three genotyping scenarios in WW (well-watered) and WS (water-stressed) conditions water supply. SPAD, PH (plant height), SD (stalk diameter), and SDM (shoot dry matter)

Trait	Scenario	Marker	Chrm	Posi (bp)	Putative annotation	Common function
**SPAD in WW**	SNP-*Array*	*Zm00001d031759*	1	200951693	Protein shoot gravitr. 5	Zinc finger proteins-gravitropism
	GBS-B73	*Zm00001d031759*	1	200951623	Protein shoot gravitr. 5	
	SNP-*Array*	*Zm00001d026477*	10	146706556	Jasmonate ZIM-domain proten	Jasmonic acid
	GBS-B73	*Zm00001d027695*	1	11300621	Glutaredoxin-C13	
	SNP-*Array*	*Zm00001d044194*	3	221628210	MYB-related-transcription factor 97	Circadian clock
	GBS-B73	*Zm00001d018127*	5	214805832	SNW/SKI-interacting protein	
**SPAD in WS**	SNP-*Array*	*Zm00001d031759*	1	200951693	Protein shoot gravitr. 5	Zinc finger proteins-gravitropism
	GBS-B73	*Zm00001d031759*	1	200951623	Protein shoot gravitr. 5	
**PH in WW**	SNP-*Array*	*Zm00001d017978*	5	211170831	Endoglucanase	Cellulose catabolic process
	GBS-B73	*Zm00001d008952*	8	26903022	Endoglucanase	
	GBS-B73	*Zm00001d021708*	7	160911849	Pentatricopeptide repeat-containing p. chloroplastic	ABA
	GBS-Mock	*Zm00001d012719*	Unique	305928723	BZIP-transcription factor	
**PH in WS**	SNP-*Array*	*Zm00001d053809*	4	241454721	Defective in cullin neddylation protein	Ubiquitination
	GBS-B73	*Zm00001d042481*	3	169056323	Ubiquitin thioesterase OTU	
**SD in WW**	GBS-B73	*Zm00001d014899*	5	67342178	Tetratricopeptide repeat (TPR)-like superfamily	Gibberellin
	GBS-Mock	*Zm00001d001852*	Unique	583680097	Gibberellin-regulated protein 2	
**SD in WS**	GBS-B73	*Zm00001d005090*	2	157770180	Clathrin heavy chain	ABA
	GBS-Mock	*Zm00001d053262*	Unique	831219654	Calcium-dependent lipid-binding family protein	
**SDM in WW**	SNP-*Array*	*Zm00001d031445*	1	190284851	Ethylene insensitive 3-like 3 protein	Ethylene
	GBS-B73	*Zm00001d027626*	1	9405820	S-adenosyl-L-methionine-dep. methy.superf. protein	
**SDM in WS**	SNP-*Array*	*Zm00001d016786*	5	175971996	PDI-like 5-2	ABA
	GBS-B73	*Zm00001d005090*	2	157770180	Clathrin heavy chain	
